# The structured ‘low temperature’ phase of the retinal population code

**DOI:** 10.1371/journal.pcbi.1005792

**Published:** 2017-10-11

**Authors:** Mark L. Ioffe, Michael J. Berry

**Affiliations:** 1 Department of Physics, Princeton University, Princeton, New Jersey, United States of America; 2 Princeton Neuroscience Institute, Princeton University, Princeton, New Jersey, United States of America; Université Paris Descartes, Centre National de la Recherche Scientifique, FRANCE

## Abstract

Recent advances in experimental techniques have allowed the simultaneous recordings of populations of hundreds of neurons, fostering a debate about the nature of the collective structure of population neural activity. Much of this debate has focused on the empirical findings of a phase transition in the parameter space of maximum entropy models describing the measured neural probability distributions, interpreting this phase transition to indicate a critical tuning of the neural code. Here, we instead focus on the possibility that this is a first-order phase transition which provides evidence that the real neural population is in a ‘structured’, collective state. We show that this collective state is robust to changes in stimulus ensemble and adaptive state. We find that the pattern of pairwise correlations between neurons has a strength that is well within the strongly correlated regime and does not require fine tuning, suggesting that this state is generic for populations of 100+ neurons. We find a clear correspondence between the emergence of a phase transition, and the emergence of attractor-like structure in the inferred energy landscape. A collective state in the neural population, in which neural activity patterns naturally form clusters, provides a consistent interpretation for our results.

## Author summary

Neurons encoding the natural world are correlated in their activities. The structure of this correlation fundamentally changes the population code, and these effects increase in larger neural populations. We experimentally recorded from populations of 100+ retinal ganglion cells and probed the structure of their joint probability distribution with a series of analytical tools inspired by statistical physics. We found a robust ‘collective state’ in the neural population that resembles the low temperature state of a disordered magnet. This state generically emerges at sufficient correlation strength, where the energy landscape develops an attractor-like structure that naturally *clusters* neural activity.

## Introduction

The past decade has witnessed a rapid development of new techniques for recording simultaneously from large populations of neurons [1–4]. As the experimentally accessible populations increase in size, a natural question arises: how can we model and understand the activity of large populations of neurons? In statistical physics, the interactions of large numbers of particles create new, *statistical*, laws that are qualitatively different from the original mechanical laws describing individual particle interactions. Studies of these statistical laws have allowed the prediction of macroscopic properties of a physical system from knowledge of the microscopic properties of its individual particles [5]. By exploiting analogies to statistical physics, one might hope to arrive at new insights about the collective properties of neural populations that are also qualitatively different from our understanding of single neurons.

The correlated nature of retinal ganglion cell spike trains can profoundly influence the structure of the neural code. Information can be either reduced or enhanced by correlations depending on the nature of the distribution of firing rates [6], the tuning properties of individual neurons [7], stimulus correlations and neuronal reliability [8, 9], the patterns of correlations [10], and interaction among all these factors [11]. In addition, the structure of the decoding rule needed to read out the information represented by a neural population can be strongly influenced by the pattern of correlation regardless of whether it reduces or enhances the total information [12, 13].

One approach to understanding the properties of measured neural activity is to study the nature of minimally structured (‘maximum entropy’) models of the probability distribution that reproduce the measured correlational structure [14–16]. These models have been shown to be highly accurate in reproducing the full statistics of the activity patterns of small numbers of neurons [15, 17, 18]. The hope is that even if these models underestimate the real structure of larger neural populations, the properties of the distribution which arise in these simplified models are general and of consequence to the true distribution.

Maximum entropy models that constrain only the pairwise correlations between neurons are generalized versions of the Ising model, one of the simplest models in statistical physics where collective effects can become significant. The macroscopic behavior of these models varies substantially depending on the parameter regime. By fitting these models to measured neural acitivity, we can begin to explore (by analogy) the ‘macroscopic’ properties of the retinal population code. In particular, we can gain insight into these macroscopic properties by introducing a fictitious temperature variable into the maximum entropy model. By changing this temperature, we can continuously tune the population from a ‘high temperature’ regime where correlation has a minimal effect on the probability distribution to a ‘low temperature’ regime where correlation dominates. Furthermore, we expect to see signatures of a phase transition at the boundary between these regimes. Thus, by observing a phase transition at a particular value of the temperature variable, we can determine if the real state of the neural population more closely resembles the high or low temperature state.

One macroscopic property of interest is the specific heat. Discontinuity or divergence of this property is an indicator of a second-order phase transition, which implies a qualitative change in the properties of the system. Previous studies have shown that the specific heat has a peak that grows and sharpens as more neurons are simultaneously analyzed [19–22]. Most of the literature on this topic can be divided into two camps: the ‘proponents’ who argue that this is a signature of criticality, i.e. the system is poised in between high and low temperature phases, in a manner that might optimize the capacity of the neural code [19, 21, 23], and the ‘sceptics’ who argue that this is merely a consequence of ignored latent variables [24, 25], ignored higher order correlation structure in the data [26], or even the presence of any correlation at all [27].

An alternative interpretation is that system is in a ‘low temperature’ state, and that the peak in the heat capacity is a signature of a first-order phase transition. The difference between the two types of transitions rests on which macroscopic properties of the system are discontinuous at the transition: first-order phase transitions have discontinuities in the entropy (hence having an infinite heat capacity), while second-order phase transitions will have discontinuities, or integrable divergences, in the specific heat. The observed sharpening of the specific heat is influenced by finite-size effects which could be consistent with either a delta function (first-order) or a divergence (second-order) in the specific heat. In principle, one can use finite-size scaling arguments to argue that the sharpening is more or less consistent with one of the two possibilities. In practice however we do not think that we can convincingly distinguish between these two possibilities with our analysis of the specific heat, and so we cite other forms of evidence in favor of our interpretation.

Empirically, the peak of the specific heat is found at a higher temperature than the operating point of the real system (*T* = 1), suggesting that the system is on the low temperature side of the phase transition. Low temperature phases in statistical physics are usually associated with structure in the distribution of states, in which the system can ‘freeze’ into ordered states. High temperature phases, in contrast, are associated with weakly correlated, nearly independent structure in the population of neurons. From this perspective, the phase transition that many studies have observed as a function of an imposed temperature variable, *T*, serves as an indicator of structure in the probability landscape of the neural population at the real operating point (*T* = 1).

Maximum entropy models fit particular statistics of the distributions of experimentally measured neural neural activity [16]. Because retinal responses are specific to both the adaptational state of the retina and the ensemble of stimuli chosen to probe them, the measured pattern of neural correlation—and hence the detailed properties of the maximum entropy model—will also vary. Therefore, it is yet unclear how robust is the presence of a low temperature state to different experimental conditions. The phase transition itself arises as a consequence of the correlations between neurons. This pattern of correlation in turn has contributions from correlations in the stimulus and from retinal processing. The distribution of correlations also has a particular shape, with many weak but statistically non-zero terms. It is unclear how these different properties contribute to the nature of the structured collective state of the neural population.

Here, we show that while the detailed statistics of the retinal population code differ across experimental conditions, the observed phase transition persists. We find that retinal processing provides substantial contributions to the pattern of correlations among ganglion cells and thus to the specific heat, as do the many weak but statistically non-zero correlations in the neural population. We also find that the spatio-temporal processing of the classical receptive field is not sufficient to understand the collective properties of ganglion cell populations. To address the nature of the collective state of the retinal population code, we explored how a particle representing the state of neural activity moves over the system’s energy landscape under the influence of finite temperature. We find that the energy landscape has regions that “trap” particle motion, in analogy to basins of attraction in a dynamical system. By varying the overall correlation strength, we show that the emergence of this structure is closely connected to the emergence of the measured phase transition. This emergence occurs at surprisingly low overall correlation strength, indicating that the real population is robustly within the structured regime.

## Results

One of the main goals of our study is to test whether the collective state of a neural population is robust to different experimental conditions. Adaptation to the statistics of the visual input is a central feature of retinal processing, and any robust property of the retinal population code should be present in different adaptational states. We focused first on adaptation, and in particular we chose an experiment probing adaptation to ambient illumination.

To process the visual world, the retina has to adapt to daily variations of the ambient light level on the order of a factor of a hundred billion [28]. Prominent examples of known sites in the retinal circuit with adaptive mechanisms include the voltage-intensity curves of photoreceptors [29–31], the nonlinear output of bipolar cells [32], and the surround structure of ganglion cells [33]. A significant contribution to these effects arises from the light-dependent global dopamine signal [34, 35], which regulates retinomotor changes in the shapes of photoreceptors [36], and gap junction transmission in horizontal [37] and AII amacrine cells [35]. The global nature of the dopamine signal suggests that any cells or synapses in the retina that possess dopaminergic receptors (almost all retinal cell types studied, [38]), will experience adaptive effects of changes in the mean light level. Though the literature on single cell adaptation to ambient illumination is extensive, little is known about the changes in correlational structure across the population of retinal ganglion cells.

We recorded from a population of tiger salamander (*Ambystoma Tigrinum*) retinal ganglion cells responding to the same natural movie at two different ambient light levels. In Experiment #1, we recorded with and then without an absorptive neutral density filter of optical density 3 (which attenuates the intensity of light by a factor of 10^3^) in the light path of the stimulus projecting onto the retina. The stimuli consisted of a chromatic checkerboard and a repeated natural movie (details in Methods). Thus, the contrast and statistics of each visual stimulus were the same under both luminance conditions, with the only difference being that in the *dark* condition, the mean light level was 1000 times lower than in the *light* condition.

**Fig 1:**
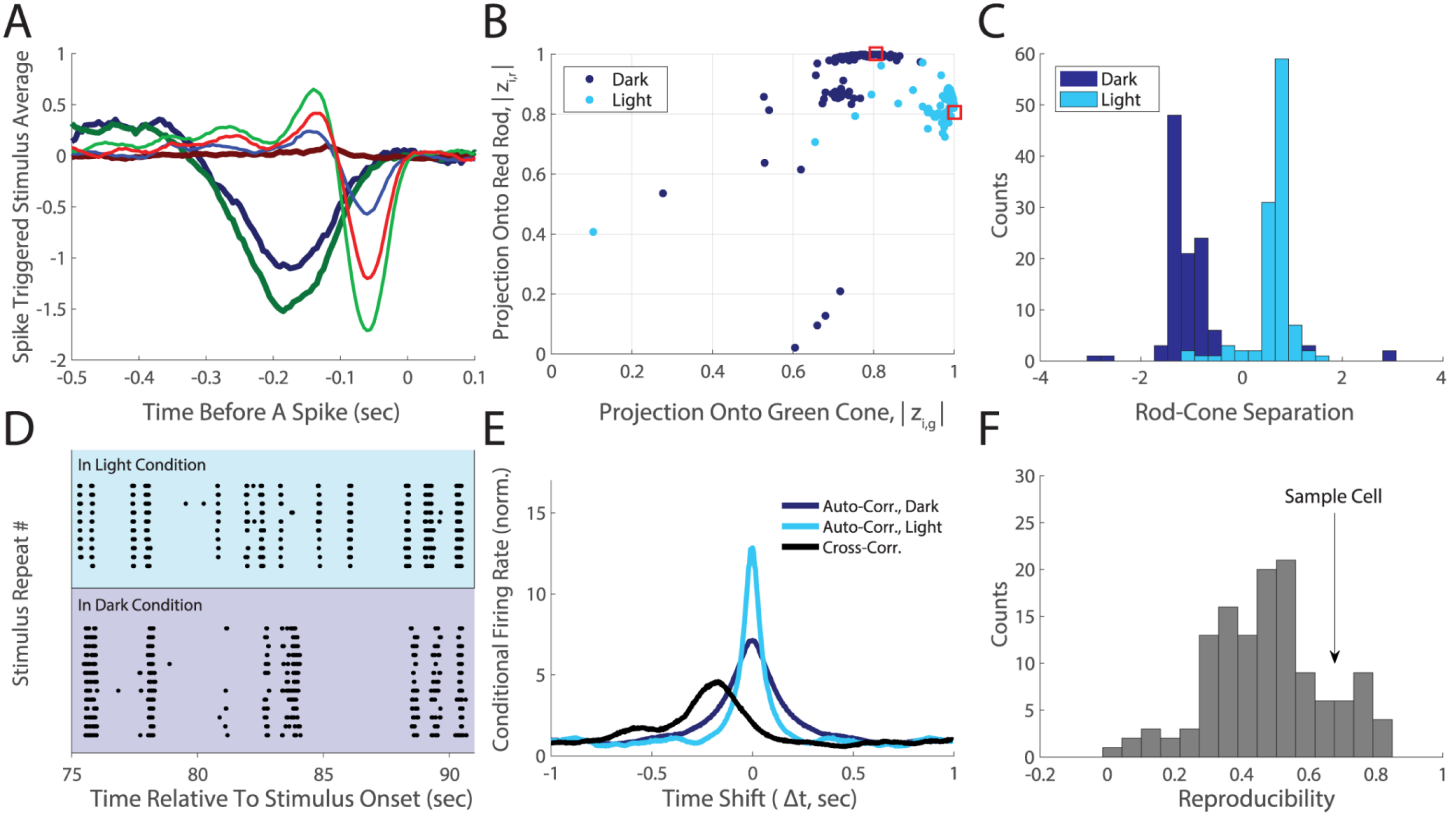
Changes in individual RGC feature selectivities are consistent with a switch between rod and cone dominated circuitry. **A**. Color-dependent STA for a sample ganglion cell (Experiment #1). Lighter shades measured in the *light* condition. Red, green and blue colors correspond to the monitor gun. **B**. Projections of the ganglion cell color profiles onto the red rod (*z*_*i*,*r*_) on the y-axis plotted versus the projection onto the green cone (*z*_*i*,*g*_) for *N* = 111 ganglion cells (see Supplement); dark blue dots for the *dark*, light blue dots for *light* condition. Absolute values are used to provide invariance to the sign of the color vector (ON- vs. OFF-type cells). The rod-cone overlap (similarity of the two photopigments) is plotted as red squares. **C**. The distribution of the rod-cone spectral separations (see Supplement), plotted for the *dark* (dark blue) and *light* (light blue) conditions. **D**. Responses of a sample ganglion cell to repeated presentations of the same stimulus segment, in the two conditions. **E**. Auto- and cross-correlations of spike trains for the cell in panel **D**, calculated excluding spikes from the same trial; *dark-dark* auto-correlation (dark blue), *light-light* auto-correlation (light blue), *dark-light* cross-correlation (black). These are normalized by the probability of a spike, so that this measure tends to 1 at long time shifts. **F**. Distribution over ganglion cells of the reproducibility (see main text) of spikes across light adapted conditions, with an arrow indicating the sample cell in **D,E**.

The responses of individual cells to the checkerboard allowed us to measure the spatiotemporal receptive field of each ganglion cell using reverse correlation [39, 40] in each light condition. For most cells these three linear filters were scaled versions of each other, suggesting a single filter with different sensitivities to the red, green and blue monitor guns (Fig 1A). The vast majority of rods in the tiger salamander retina are classified as ‘red rods’ (98%); similarly, most of the cones are ‘green cones’ (80%) [41, 42]. We estimated the relative sensitivities of these two photopigments to our monitor guns from the reported spectra of these photopigments [42–44] and a measurement of the spectral output of the monitor guns (see Supplement). We found that for many ganglion cells the relative amplitudes of these three sensitivities were closely consistent with the estimated sensitivity of the red rod photopigment in the *dark* recording, and the green cone photopigment in the *light* recording (Fig 1B and 1C). These results suggested to us that in our experiments, retinal circuitry was in the scoptopic, rod-dominated limit in our *dark* condition and in the photopic, cone-dominated limit in our *light* condition.

How much does the feature selectivity of ganglion cells change across the two light-adapted conditions? We can see from the temporal kernel of the spike-triggered average that there are some changes in temporal processing along with a big change in response latency (Fig 1A). Another way to compare feature selectivity is to look at what times a ganglion cell fires spikes to repeated presentations of the same natural movie (Fig 1D). The spike timing of individual ganglion cells was reproducible across repeats of a natural movie within a particular luminance condition. However, across conditions there were significant changes in which times during the stimulus elicited a spike from the same cell (Fig 1D).

To quantify this effect over our entire recording, we estimated the shuffled autocorrelation function of each cell’s spike train (i.e. the correlation function between spikes on one trial and those on another trial [45]). The width of this shuffled autocorrelation function is one measure of timing precision for the spike train [45]. The narrower width of the autocorrelation curve in the *light* adapted condition indicated greater spike timing precision in that condition. The 200ms offset in the peak of the cross correlation curve was characteristic of the longer delays in signal processing that arise in the rod versus cone circuitry, which can also be seen in the different latencies of the reverse correlation (Fig 1A). The area under the curve, but above the random level set by the firing rate, is a measure of reproducibility across trials. Normalizing this measure across luminance conditions yielded an estimate of spiking reproducibility across light conditions. A value of unity for this measure indicates that spiking reproducibility across light conditions is as high as the reproducibilities within each condition. We found a wide range of reproducibility across our populations of neurons (0 to 0.75, Fig 1E), suggesting that significant changes in feature selectivity occured for most neurons. Our results are qualitatively consistent with a recently published study [46], where retinal ganglion cells gain or lose specific firing events at different ambient light levels.

Given that our observed changes in chromatic sensitivites and feature selectivities of ganglion cells were consistent with distinct adaptational states for the retina, it seemed likely that we would find statistically significant changes in the average firing rates, pairwise correlations, and the distribution of simultaneously active cells. Because the details of the maximum entropy model are a function of this set of constraints, statistically significant changes in these moments across light conditions were particularly important. Otherwise, any observed ‘robustness’ at the collective level would be trivial.

**Fig 2:**
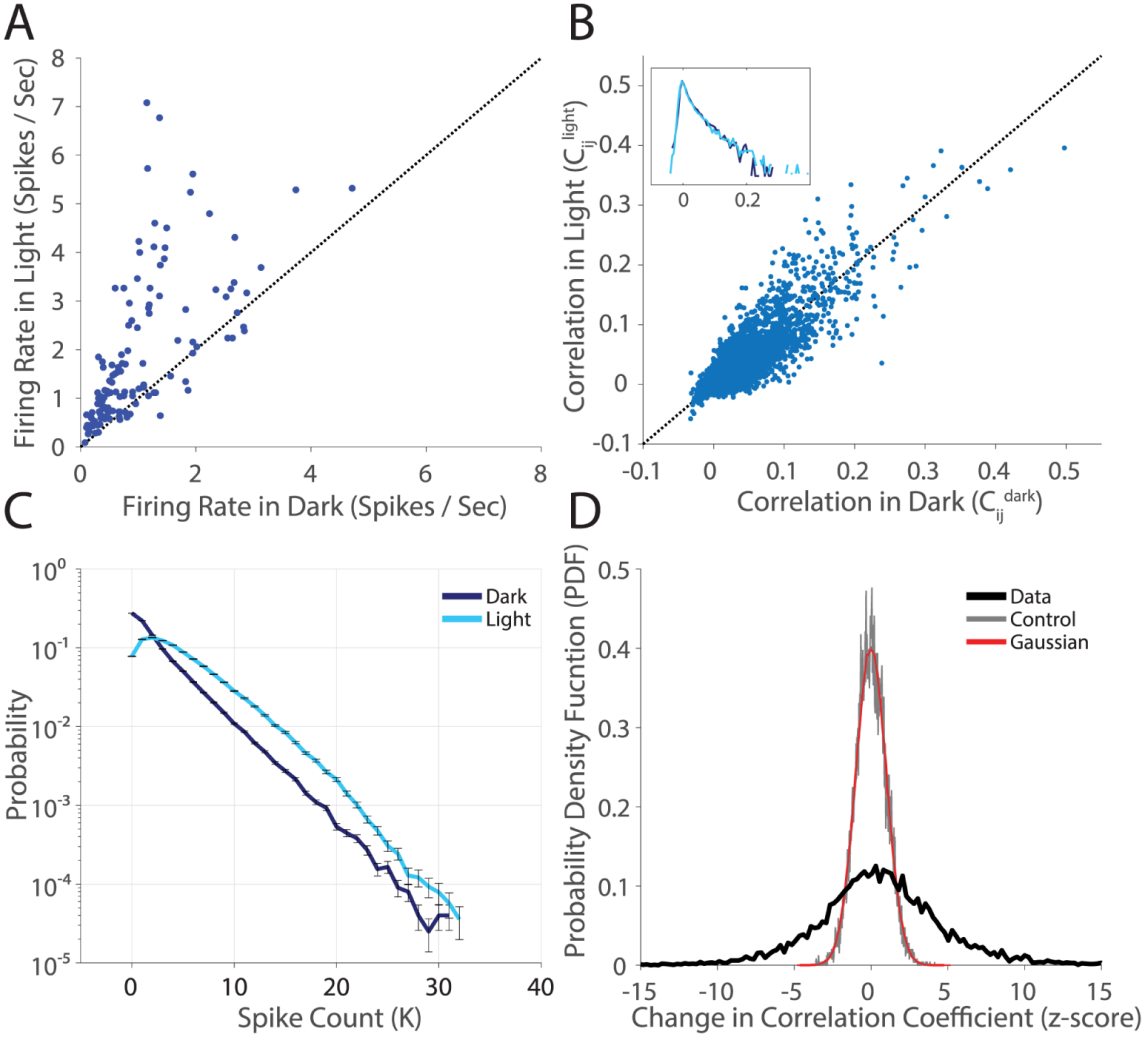
Statistically significant changes in population structure across stimulus conditions. **A**. Firing rate in the *light* condition plotted against firing rate in the *dark* for all *N* = 128 cells. Error bars are given by the standard error of the mean (SE) and are smaller than the plotted points. **B**. Pairwise correlation in the *light* plotted against pairwise correlation in the *dark*, for all 128 ⋅ 127/2 pairs of cells. Error Bars are not shown (but see panel **D**). Inset is the probability density function (PDF), on a log scale, of correlation coefficients. **C**. Measured P(K) in the two light conditions. *K* is the number of active cells in a state. Error bars are given by the SE. **D**. The PDF of z-scores of changes in correlation coefficients. The change in correlation coefficient is normalized by the error ($\sigma =\sqrt{\sigma_{d}^{2}+\sigma_{l}^{2}}$). These error bars are standard deviations over bootstrap resamples of the data, estimated per cell pair. Data compares the *light* and *dark* adapted conditions (thick black line), the control compares a random half of the dark dataset to the other half (gray), and a numerical gaussian is plotted in red for comparison.

We found that firing rates mostly increased at the higher light level, an effect that was highly statistically significant (Fig 2A). The correlation coefficients between pairs of cells showed some increases and some decreases between the *light* and *dark* adaptational states (Fig 2B). Overall, the distribution of correlation coefficients was roughly the same. However, the detailed pattern of correlation appeared to change. To evaluate the significance of changes in correlation coefficients, we estimated the difference between the correlation coefficients in the two light-adapted conditions normalized by the error bar—a measure also known as the z-score. The distribution of z-scores across all pairs of ganglion cells (Fig 2D, black curve) had significant density in the range of 5 − 10 standard deviations, a result that is not consistent with controls (random halves of the *dark* dataset compared again each other, gray curve), or with the curve expected for the null hypothesis (a Gaussian with standard deviation of one, red curve). Thus correlation coefficients between individual pairs of cells change far more than expected by chance. The final ingredient in the maximum entropy model, the probability of *K* simultaneously active cells, (*P(K*)), displayed a statistically significant overall shift towards sparseness in the *dark* adapted condition (Fig 2C).

Following previously published results [16, 19], we modeled the distributions of neural activity with the k-pairwise maximum entropy model, which approximates the probability of all patterns of activity in the ganglion cell population. Here, we binned each ganglion cell’s spike train in 20 ms time windows, assigning 0 for no spikes and 1 for one or more spikes, *r*_*i*_ = [0, 1]. We denote a particular population activity pattern as *R* = {*r*_*i*_}. The probability of state *R* in this model is given by:

$$\begin{array}{c} P\left(R\right)=\frac{1}{Z}\exp\left(-E_{k-\text{pairwise}}\left(R\right)\right) \end{array}$$ (1)

where *Z* is the normalizing factor, and we’ve introduced a unitless ‘energy’:

$$\begin{array}{c} E_{k-\text{pairwise}}\left(R\right)=\sum_{i}^{N}h_{i}r_{i}+\sum_{i,j\neq i}^{N}J_{ij}r_{i}r_{j}+\sum_{k=1}^{K}\delta (\sum_{i}r_{i},k)\lambda_{k} \end{array}$$ (2)

with *δ* the Kronecker delta. The shapes of the probability and energy landscapes have a one-to-one relationship to each other, with energy minima corresponding to probability maxima. Because of the extensive intuition surrounding the concept of an energy landscape in physics, we will often use this term. This model is constrained to match the expectation values 〈*r*_*i*_〉, 〈*r*_*i*_*r*_*j*_〉 and *P(K)* measured in the data. We inferred these models with a modified form of sequential coordinate descent [16, 47, 48] [see also Methods, S2, S3, S4 and S5 Figs].

### The collective state of a neural population

A first step towards the study of collective phenomena in neural populations is to understand what is the qualitative nature or ‘phase’ of the neural population. Phases of matter occur everywhere in nature where there is some collective structure in the population. In the theory describing phase transitions in statistical physics, first-order phase transitions can occur when a particular system can decrease its free energy by transitioning to a new phase. While such a transition in our work here will occur in a region of parameter space that is not real—i.e., is not visited by the retina experimentally—its occurence provides evidence for structure in the real experimentally measured distribution. Thus, an explanation of previously observed phase transitions is that the pattern of correlation among ganglion cells induces a highly structured phase which is qualitatively different from the phase found in the high temperature limit. From this perspective then, we ask whether this phase is robust to different adaptational states, and what are the properties of the retinal population code that give rise to that phase?

**Fig 3:**
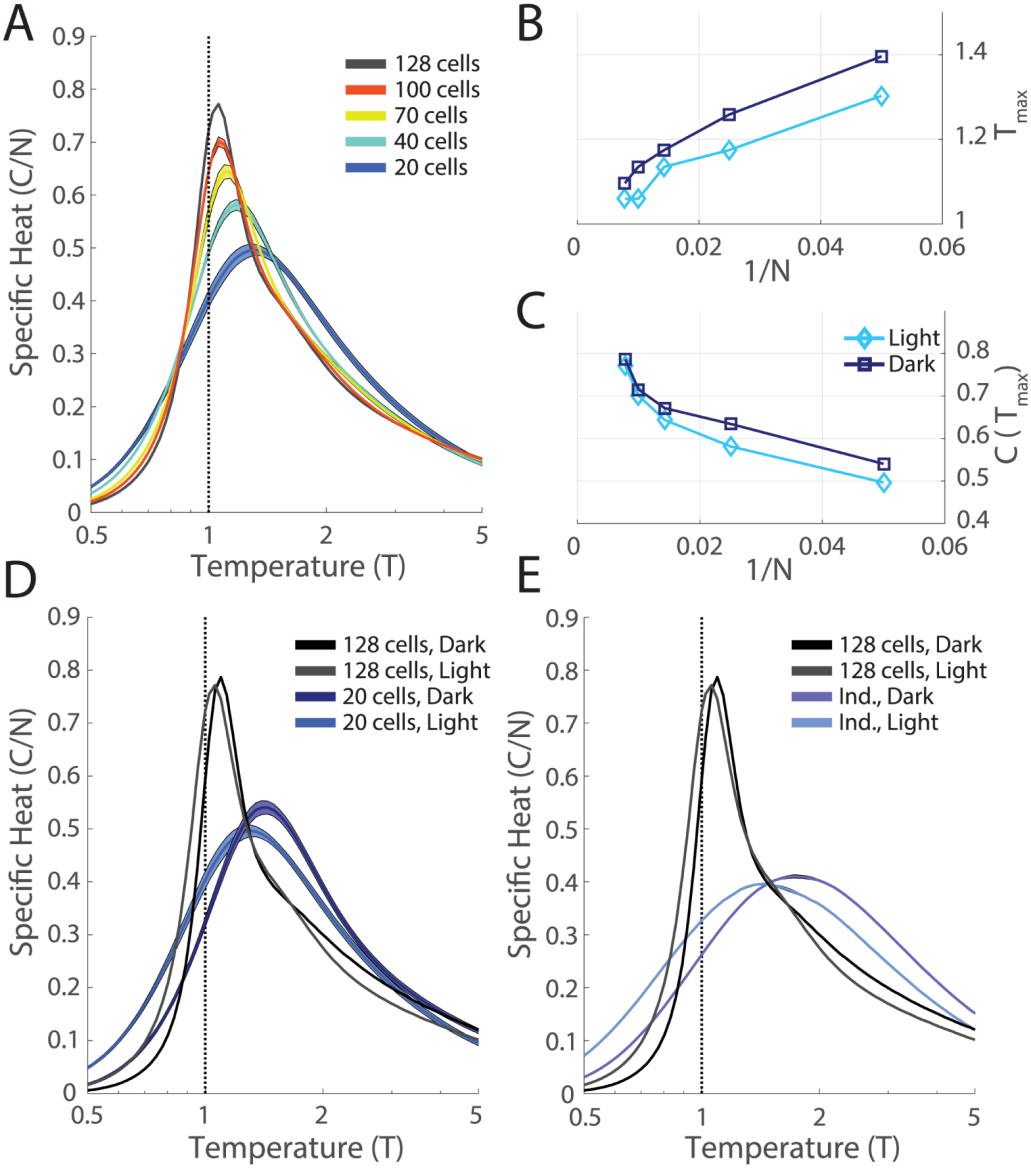
Phase transitions robustly present in both stimulus conditions. **A**. For each value of *N*, we selected 10 groups of cells (out of the *N* = 128, M1, *light* recording). For each group we inferred the k-pairwise maximum entropy model as in [19], and estimated the specific heat. Shaded areas are the SE. **B,C**. Peak temperature, *T*_*max*_, and peak specific heat, *C*(*T*_*max*_), plotted as a function of inverse system size, 1/*N*. **D**. Specific heat plotted versus temperature for the full network (*N* = 128) and for smaller subnetworks (*N* = 20) for both the *dark* and *light* conditions. Error bars as in **A**. **E**. Comparison of the specific heat of the full network to that of an independent network estimated from shuffled data, for both the *dark* and *light* conditions.

To study the emergence of a phase transition with increasing system size, we subsampled groups of *N* neurons and inferred models for these subsets of the full neural data. For all of these networks, we then introduced a fictitious temperature parameter, *T*, into the distribution, *P*(*R*) = (1/*Z*(*T*))exp(−*E*(*R*)/*T*). This parameter allows us to visit parameter regimes of our model where the qualitative nature of the system changes. If the shape of the specific heat as a function of temperature exhibits a sharp peak, this indicates a phase transition—a macroscopic restructuring of the properties of the system across parameter regimes. Thus, an analysis where we vary the effective temperature allows us to gain insight into the state of the real neural population at *T* = 1. As previously described [19], we found a peak in the specific heat that sharpened and moved closer to *T* = 1 with increasing system size, *N* (Fig 3A–3C).

The systematic changes that we observe as a function of the system size *N* (Fig 3A–3C) indicate that correlation plays a more dominant role as the population size increases. To further understand the role of correlations, we performed a shuffle test, where we broke correlations of all orders in the data by shifting each cell’s spike train by a random time shift (including periodic wrap-around) that was different for each cell. Following this grand shuffle, we repeated the full analysis procedure described above (fitting a maximum entropy model to the shuffled data and estimating the specific heat). We found that the heat capacity had a much lower and broader peak that did not change as a function of *N*. In addition, this heat capacity curve agreed closely with the analytical form of the specific heat for an independent neural population (S6 Fig). This analysis demonstrates that the sharpening of the specific heat that we observed is a direct consequence of the measured pattern of correlation among neurons [Fig 3E].

The shuffled curves were noticeably different across light adapted conditions (Fig 3D). This is not surprising as the analytical form for the specific heat of a network of independent neurons depends only on the average firing rate of each neuron, and these are substantially different between the two luminance conditions (Fig 2A). However, the heat capacity peaks for both the *dark* and the *light* conditions became more similar with increasing *N*. Clearly some macroscopic properties of the network were conserved across luminance conditions for the real, correlated, data (Fig 3).

**Fig 4:**
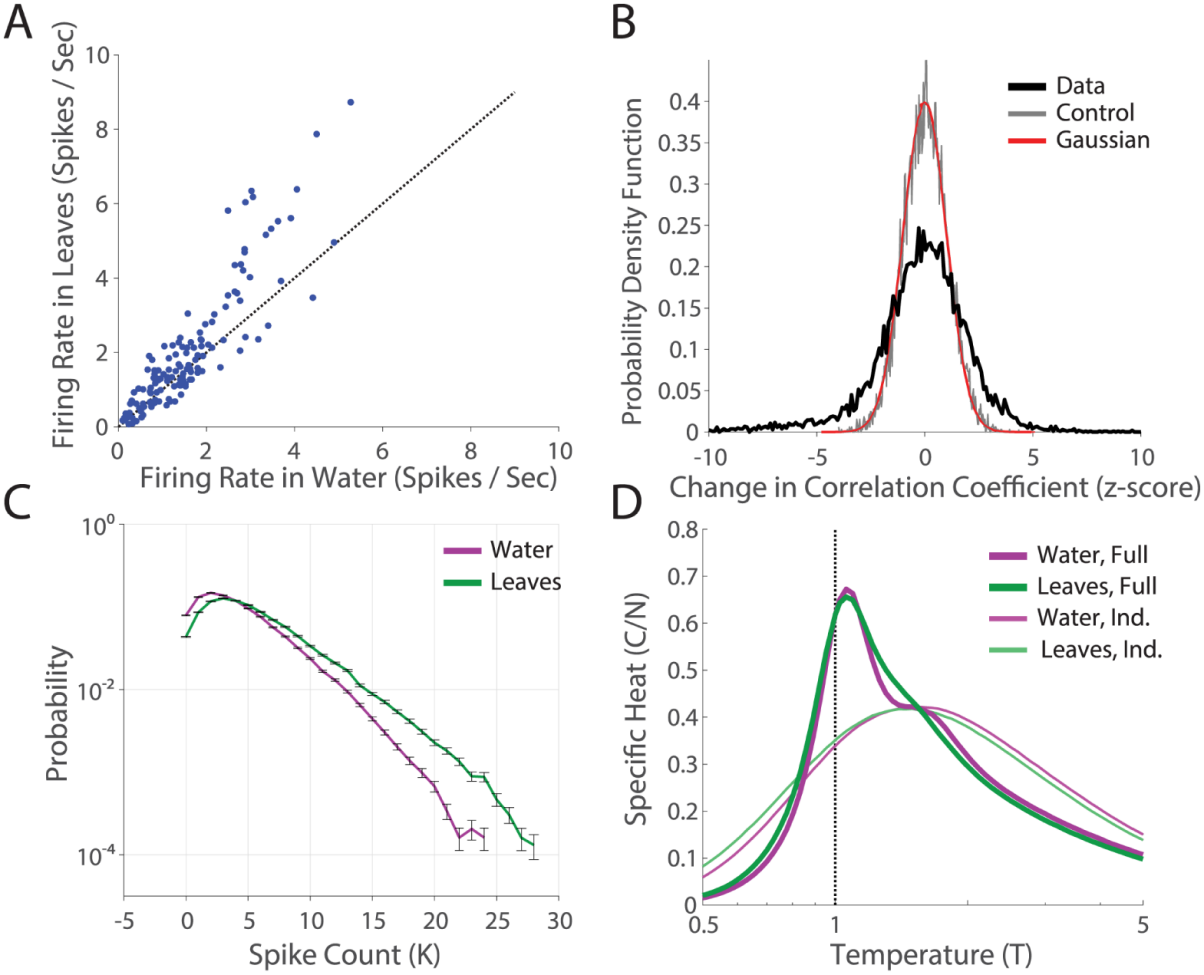
Robustness of phase transition to alternate adaptive mechanisms. **A**. Firing rate in the “leaves” natural movie (M1) plotted against firing rate in the “water” natural movie (M2) condition, for all *N* = 140 cells. Error bars are the SE. **B**. The distribution of changes in correlation coefficient across the two stimulus conditions, for all 140 ⋅ 139/2 pairs of cells. **C**. Distributions of the spike count, P(*K*), across the two stimulus conditions. **D**. Specific heats for full data and shuffled data (calculated as in Fig 3), in the two natural movie conditions.

The correlation structure of natural movies can in principle trigger a broad set of observed retinal adaptation mechanisms, such as adaptation to spatial contrast [49, 50], temporal contrast [51], and relative motion of objects [52]. To generalize our results to these higher-order adaptive mechanisms, we ran another experiment comparing the distributions of responses of the same retina to two different natural stimuli ensembles, without a neutral density filter (Experiment #2, see Methods). These two natural movies were of grass stalks swaying in the wind (M1, the same movie as in the previous experiment), and ripples on the surface of water near a dam (M2). The first movie (M1) had faster movements, larger contrasts, and fewer periods of inactivity. Likely as a consequence, we found higher firing rates in ganglion cells during M1 (Fig 4A). We found statistically significant differences in the correlation coeffecients, *C*_*ij*_, and *P(K)* across the two stimulus conditions (Fig 4B and 4C). However, the specific heats of the full networks in the two movies sharpened similarly across conditions (Fig 4D), indicating that this macroscopic property of the retinal population code was also robust to different choices of naturalistic stimuli.

### The role of retinal processing for the collective state

So far, our results have demonstrated that the peak in the specific heat is due to the pattern of correlation among neurons. However, these correlations have contributions both from retinal processing, such as the high spatial overlap between ganglion cells of different functional type [53, 54], and from the correlation structure in the stimulus itself. In order to compare the relative importance of these two different sources of correlation among ganglion cells, we measured neural activity during stimulation with a randomly flickering checkerboard. By construction, our checkerboard stimulus had minimal spatial and temporal correlation: outside of 66 *μm* squares and 33 ms frames, all light intensities were randomly chosen. Returning to Experiment #1 in the light-adapted condition, we compared the response of the retina to the natural movie and the checkerboard stimuli (Note that here we are working with the *N* = 111 ganglion cells that were identifiable across both conditions, a subset of the *N* = 128 ganglion cells we worked with in Fig 3).

**Fig 5:**
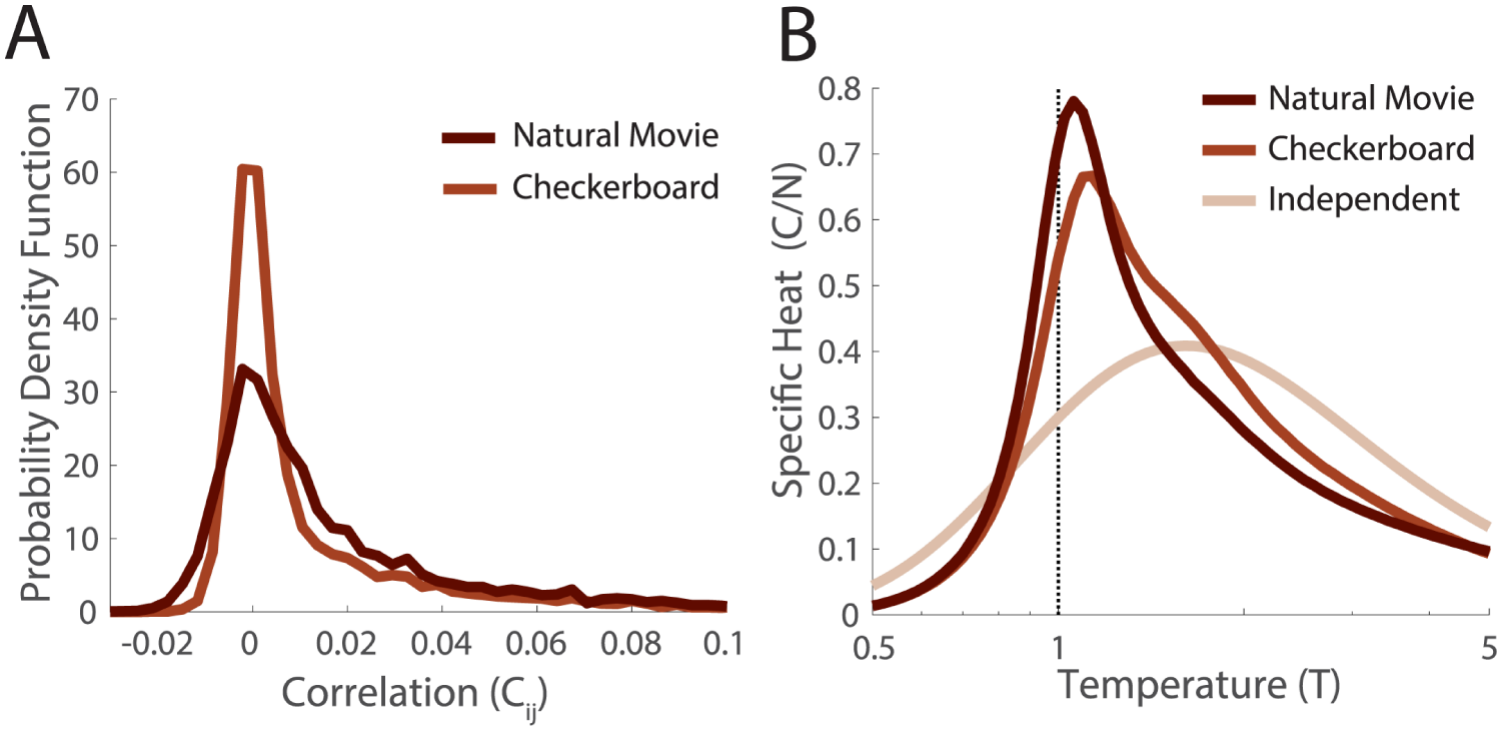
Dependence on correlation structure of naturalistic stimuli. **A**. Distributions of correlation coefficients in the naturalistic and artificial stimulus conditions (Experiment #1, *light*, *N* = 111 ganglion cells). **B**. Specific heats in the naturalistic and artificial stimulus conditions. Here the independent curve was calculated analytically based on the firing rates in the checkerboard condition.

The distribution of pairwise correlation coefficients was tighter around zero when the population of ganglion cells was responding to the checkerboard stimulus (Fig 5A). The specific heat in the checkerboard was smeared out relative to the natural movie, but was still very distinct from the independent population (Fig 5B). This suggested to us that most, but not all, of the contributions to the shape of the specific heat were shared across the two stimulation conditions, and therefore arose from retinal processing.

A simple and popular view of retinal processing is that each ganglion cell spike train is described by the spatio-temporal processing of the cell’s classical receptive field. In this picture, correlation between ganglion cells arises largely from common input to a given pair of ganglion cells which can be described by the overlap of their receptive fields. To explore the properties of this simple model, we estimated linear-nonlinear (LN) models for each of the *N* = 111 ganglion cells in the checkerboard recording (Methods). We then generated spike trains from these model neurons responding to a new pseudorandom checkerboard sequence, and binarized them into 20ms bins in the same manner as for the measured neural data. As expected, the receptive fields had a large degree of spatial overlap [53, 55], which gives rise to significant stimulus-dependent correlations.

**Fig 6:**
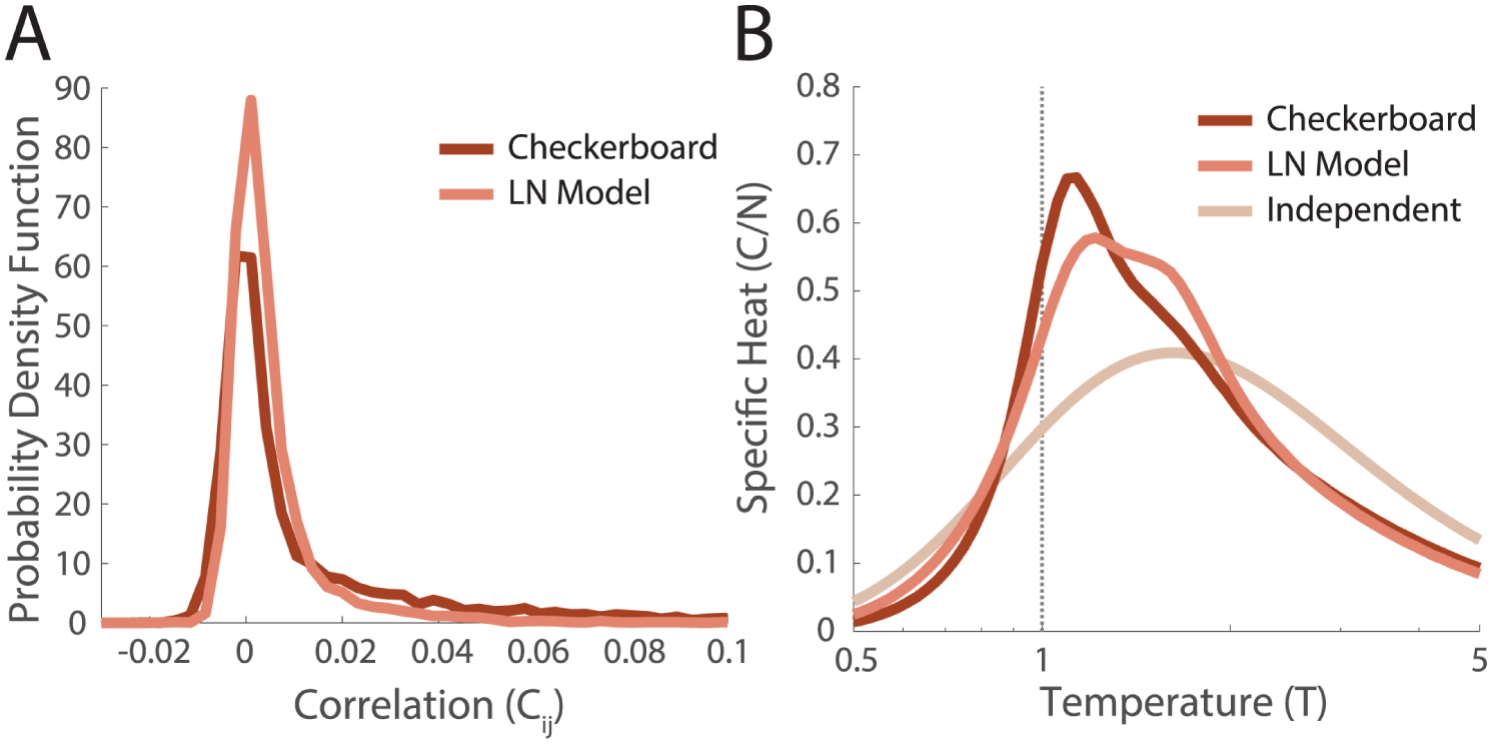
Networks of model LN neurons. Model LN neurons were fit to the measured receptive fields as described in the text, *N* = 111 ganglion cells. **A**. Distributions of correlation coefficients over pairs of cells estimated in the training data (responding to checkerboard), and the simulated network of LN neurons. **B**. The specific heats in the checkerboard and simulated LN network. The independent curve is the same analytic estimate as in Fig 5.

We found that these networks did not reproduce the distributions of correlations found in the data, instead having lower values of correlation and fewer outliers (Fig 6A). The specific heat of the network of LN neurons was reduced relative to the neural data that the LN models were based upon (Fig 6B). Thus, the peak in the specific heat is enhanced by the nonlinear spatial and temporal computations in the retina that are not captured by models of the classical receptive field.

### What pattern of correlation is needed for the collective state?

Because the detailed properties of the maximum entropy model depend strictly on correlations measured within the neural population, we wanted to develop a more general understanding of what aspects of the pattern of correlation were essential. To do this, we altered particular properties of the measured matrix of correlations, keeping the firing rates constant. We then inferred the maximum entropy model parameters for these new sets of constraints, and estimated the specific heat. For these manipulations, we worked with the simpler pairwise maximum entropy model. We made this choice for several reasons. First, manipulating only the pairwise correlation matrix made our analysis simpler and more elegant than also having to perturb the distribution of spike counts, *P(K)*. There is a large literature reporting values of pairwise correlation coefficients, helping us to make intuitive choices of how to manipulate the correlation matrix, while very little such literature exists for *P(K)*. Additionally, any perturbation of the correlation matrix consequently changes *P(K)*, so that attempting to change the correlation matrix while keeping *P(K)* fixed is a nontrivial manipulation. Second, in the pairwise model all effects of correlational structure are confined to the interaction matrix. This interaction matrix has been studied extensively in physics [56], and hence there is some intuition as to how to interpret systematic changes in the parameters. Conversely, we have little intuition currently for the nature of the k-potential. Our final and most important reason was that the qualitative behavior in the heat capacity (sharpening with system size, convergence across light and dark datasets) is the same for both pairwise and k-pairwise models across all conditions tested (S7 Fig).

**Fig 7:**
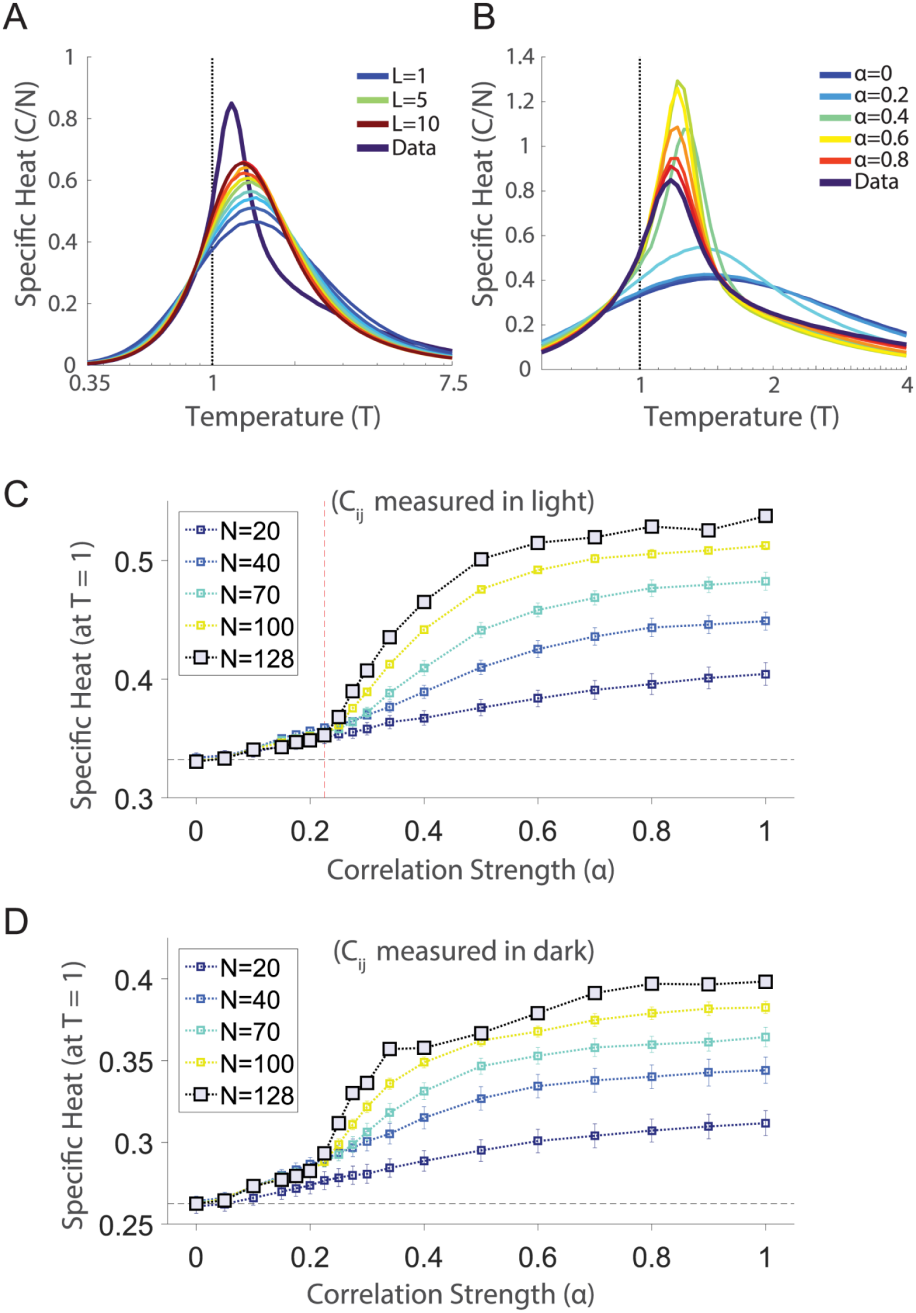
Targeted manipulations of the correlation matrix. **A**. The specific heat plotted versus temperature for several values of the connectivity, *L* (see text). **B**. The specific heat plotted versus temperature for several values of the global covariance *α* (see text). **C**. The specific heat at the operating point of the network (*T* = 1) plotted as a function of *α*. For subnetworks of different sizes, we inferred the pairwise maximum entropy models for a given *α*. Error bars are standard error of the mean over ten different choices of cells per subnetwork size. Red dashed line indicates our estimate of the discontinuity (which occurs between the values of *α* = 0.225 and *α* = 0.25). **D**. Same calculation as **C**, but for correlations and firing rates measured in the *dark*.

The correlation matrix in the retinal population responding to a natural stimulus has many weak but statistically non-zero correlations [54, 55], a result also found elsewhere in the brain [57, 58]. To test their contribution to the specific heat, we kept only the largest *L* correlations per cell, replacing the other terms in the correlation matrix with estimates from the shuffled (independent) covariance matrix. If our results are based on a “small world network” of a few, strong connections [59], then the specific heat for small values of *L* should begin to approximate our results for the real data. Clearly (Fig 7A), even keeping the top *L* = 10*N* (out of a total of *L* = 63.5*N* values) strongest correlations did not reproduce the observed behavior. Therefore, the full “web” of weak correlations contributed substantially to the shape of the specific heat of the retinal population code.

We were next interested in understanding the qualitative nature of how networks transition between the independent and fully correlated regimes. Our approach was to scale all the pairwise correlations down by a constant (*α*), to subselect groups of neurons as previously in Fig 3, and to follow the inference procedure described above. Specifically, we formed a new correlation matrix $C_{ij}^{\;\text{mixed}}\left(\alpha \right)=\alpha C_{ij}^{\;\text{true}}+\left(1-\alpha \right)C_{ij}^{\;\text{shuff}}$. We found that the specific heat of the neural population exhibited a transition between independent and fully correlated behavior, as the correlation strength, *α*, ranged from 0 to 1 (Fig 7B).

Peaks in the heat capacity similar to the one observed in the full model emerge when *α* is greater than a critical value α*, which we estimated to be between 0.225 to 0.25 (Fig 7B and 7C). Substantially similar behavior was observed in the *dark* condition as well (Fig 7D). These data suggest that the low temperature phase emerges near α*. In fact this behavior constitutes another phase transition which can be observed without the introduction of a fictitious temperature parameter, in the curve of the specific heat of the real system (*T* = 1) as a function of correlation strength *α* (Fig 7C). There is a clear emergence of a contribution to the specific heat that depends on the system size, *N*. This additional contribution gives rise to a discontinuity in either the specific heat or its derivative. Similar behavior is observed in a classic model of spin glasses, the Sherrington Kirkpatrick (SK) model [60], as we describe in the Discussion.

Importantly, the critical value of alpha at which we see a transition to structure, α*, was substantially smaller than the measured correlation strength, *α* = 1. This indicates that the population of retinal ganglion cells had a overall strength that was “safely” within the strongly correlated regime. Thus, the low temperature state is robust to changes in adaptational state or stimulus statistics that might shift the overall strength of correlations among neurons.

### What is the nature of the collective state?

Our hypothesis was that the emergence of a phase transition was correlated with the emergence of structure in the energy landscape. Previously, the structure of the energy landscape has been studied with zero temperature Monte Carlo (MC) mapping of local minima [16], where one changes the activity state of single neurons such that the energy of the population activity state always decreases. States from the data were thereby assigned to local minima in the energy landscape, which can be thought of as a method of clustering a set of neural activity patterns into population “codewords” [16]. If each cluster encodes a specific visual stimulus or class of stimuli, then this clustering operation provides a method of correcting for errors introduced by noise in the neural response.

There are two reasons why we chose to study the structure of the energy landscape at the operating point of the system (*T* = 1). First, when we performed zero temperature descent with our models, our primary finding was that the overwhelming majority of states descended into the silent state (only 503 out of 1.75 ⋅ 10^5^ did not descend into silence on a sample run). This indicated that the energy landscape had very few local minima. Thus we needed a different approach to explore the structure of the energy landscape. Second, we were interested in properties of the system (such as the specific heat) that were themselves temperature dependent, so it made sense to stick with the real operating point of the neural population (*T* = 1).

When analyzing sufficiently large neural populations (typically, “large” means *N* > 20 cells), there are too many states to simply ennumerate them all. As a consequence, the energy landscape was accessed indirectly, through a Markov Chain Monte Carlo (MC) sampler [61], which simulates an exploration of phase space by defining the state-dependent transition probabilities between successive states. Provided that these transition probabilities are properly defined, the distribution of samples drawn should approach the desired (true) distribution with sufficient sampling. The set of these transition probabilities across all the neurons defines a ‘direction of motion’ in neural response space. We will study these directions of motion as a way to gain more insight into the properties of the energy landscape of our measured neural populations. Note, however, that MC sampling dynamics is used here as a tool to explore the geometric properties of the probability landscape of the retinal population; these are not claims of how the real dynamics of activity states change in the retina under the influence of the visual stimulus.

To study the relationship between the directions of motion given by the MC sampling process and the observed phase transition, we returned to the manipulation with scaled covariances. For a given state *R*, the MC sampler in each model (inferred for a particular value of the correlation strength *α*) will return a vector of conditional probabilities *X*(*R*, *α*) = {*x*_*i*_}, where the conditional probability for each cell *i*’s activity is given by

$$\begin{array}{c} x_{i}\left(R,\alpha \right)=\text{exp}\left(h_{i}^{\left(\text{eff},\alpha \right)}\left(R\right)\right)/\left(\text{exp}\left(h_{i}^{\left(\text{eff},\alpha \right)}\left(R\right)\right)+1\right) \end{array}$$ (3)

with an effective field, given by

$$\begin{array}{c} h_{i}^{\left(\text{eff},\alpha \right)}\left(R\right)=h_{i}^{\left(\alpha \right)}+2\sum_{j\neq i}J_{ij}^{\left(\alpha \right)}r_{j} \end{array}$$ (4)

**Fig 8:**
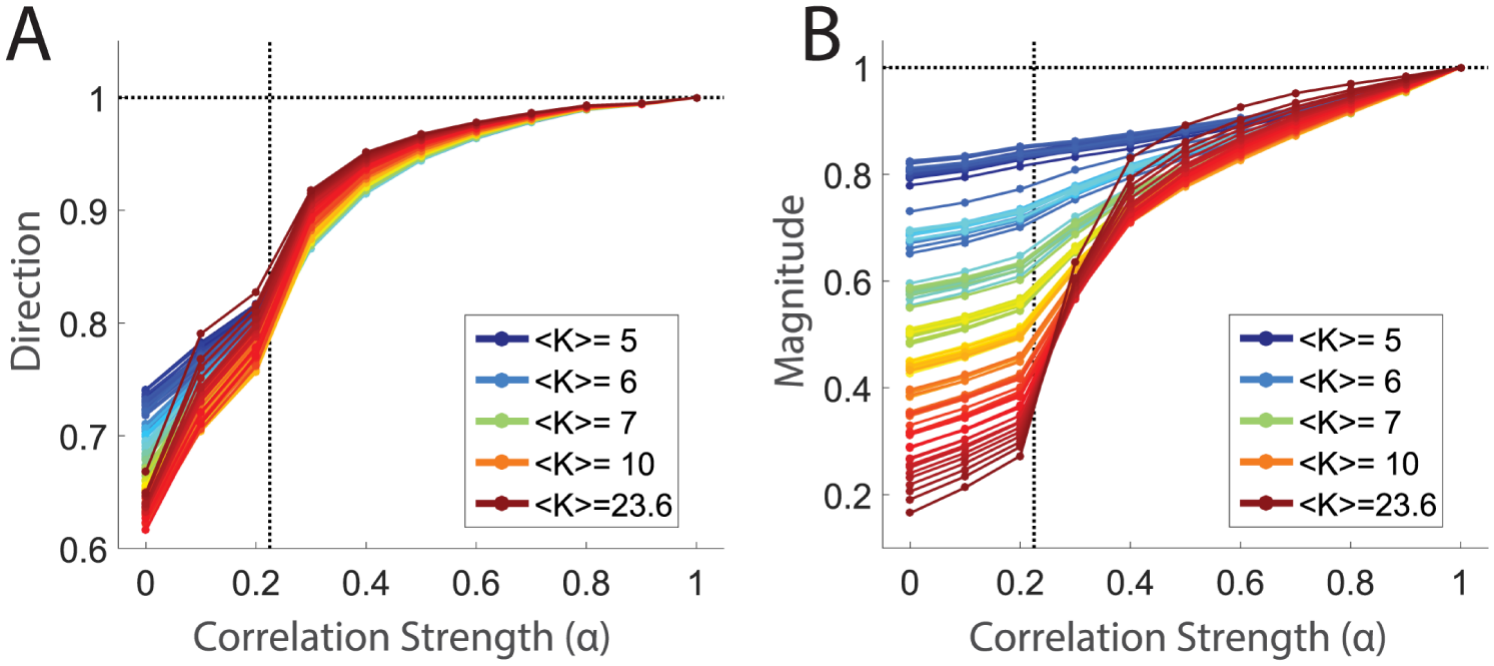
Emergence of structure in the energy landscape at low covariance strength. All states in the *light* dataset with five or more spike counts were sorted by spike count and grouped into 100 equally populated groups. Averages were calculated over the states within each group (for clarity here indexed by *p*); the color of the line indicates the average spike count within a group (1613 states per group). **A**. Average similarity in direction of sampling between full model and model at correlation strength *α*. This is estimated as the average over data states, *p*, within a given group of states: ${\langle \;\;\hat{X}\left(R_{p},\alpha \right)\cdot \hat{X}\left(R_{p},\alpha =1\right)\;\rangle }_{p}$. **B**. Similarities of magnitudes, estimated as 〈||*X*(*R*_*p*_, *α*)|| / ||*X*(*R*_*p*_, *α* = 1)||〉_*p*_. Dotted black lines on both panels indicate α* and unity.

We can now ask how the shape of the energy landscape evolved with respect to *α*. Specifically, we first compared the similarity in direction of these ‘Monte Carlo flow’ vectors with the vectors defined for the fully correlated model (at *α* = 1), by calculating the average overlap between states, *p*, at a correlation strength *α* with the same states at *α* = 1, $\hat{X}\left(R_{p},\alpha \right)\cdot \hat{X}\left(R_{p},\alpha =1\right)$. We found that in the independent limit, *α* = 0, the flow vectors pointed in substantially different directions than in the fully correlated population (Fig 8A). This indicates that there is very different structure in the energy landscape in these two limits. Furthermore, as alpha increased from zero, we found a steep increase in the similarity of flow vector directions until a little past our estimate of the critical value, *α* ≈ 0.3, at which point the slope became more and more shallow. The probability landscape roughly settled into its fully correlated ‘shape’ by *α* ≈ 0.5, which was comparable with the point by which the specific heat had stabilized near its fully correlated value as well (Fig 7C). This is consistent with a tight connection between the emergence of a phase transition and the development of structure in the probability landscape.

We carried out a similar analysis to compare the amplitude of Monte Carlo flow vectors as a function of the correlation strength, *α* (Fig 8B). At *α* = 0, we found that flow vector magnitudes were very different from the fully correlated population for states with high spike count, *K*. The similarity in amplitude increased gradually up to α*, increased sharply from α* up to *α* ≈ 0.5, and then changed slowly at higher values of *α*. Again, these results are consistent with the interpretation that the shape of the energy landscape emerged at a correlation strength near α* and that further increases in *α* served to ‘deepen’ the existing contours in the energy landscape.

While our energy landscape does not have many true local minima, we can gain insight into the nature of the energy landscape induced by correlations by considering how long the system remains in the vicinity of a given state under *T* = 1 MC dynamics. Since the experimentally measured neural activity is sparse, the directions of motion are heavily biased towards silence. Regardless of initial state, the sampler will eventually revisit the silent state.

To demonstrate this, we returned to the data from Experiment #1 (M1, *light*), and the corresponding k-pairwise model fits. Due to the addition of the k-potential in this analysis, the effective field was now

$$\begin{array}{c} h_{i}^{\left(\text{eff}\right)}\left(R\right)=h_{i}+2\sum_{j\neq i}J_{ij}r_{j}+\lambda_{K+1}-\lambda_{K} \end{array}$$ (5)

with *K* = ∑_*j*≠*i*_ *r*_*j*_.The effective field is derived as the difference in energy of the system when cell changes from silent to spiking (Eq 3), and so it has three contributions: one from the local field, *h*_*i*_, another from the sum of pairwise interactions, and a final term from the change in the k-potential, *λ*_*K*+1_ − *λ*_*K*_. We worked with all the states observed in the *light* condition that had *K* = 12 spiking cells (total number of states = 3187). We selected a set of initial activity states all having the same value of *K*, as these analyses depended strongly on *K*. We wanted a value of *K* large enough that effective fields were large, and hence collective effects of the population code were significant. At the same time, we needed *K* to be small enough that we could observe many such states in our sampled experimental data. Balancing these two concerns, we chose *K* = 12.

In order to characterize the ‘dwell time’ of a particular state, we initiated many MC sampling runs from that given state. On each of these runs, we defined the dwell time as the number of MC samples (where all N cells were updated) required to change 9 of the 12 originally spiking cells to silent. For each given initial state, our analysis produced a distribution of dwell times, due to the randomized order of cell choice during sampling, as well as the stochasticity inherent in sampling at *T* = 1.

**Fig 9:**
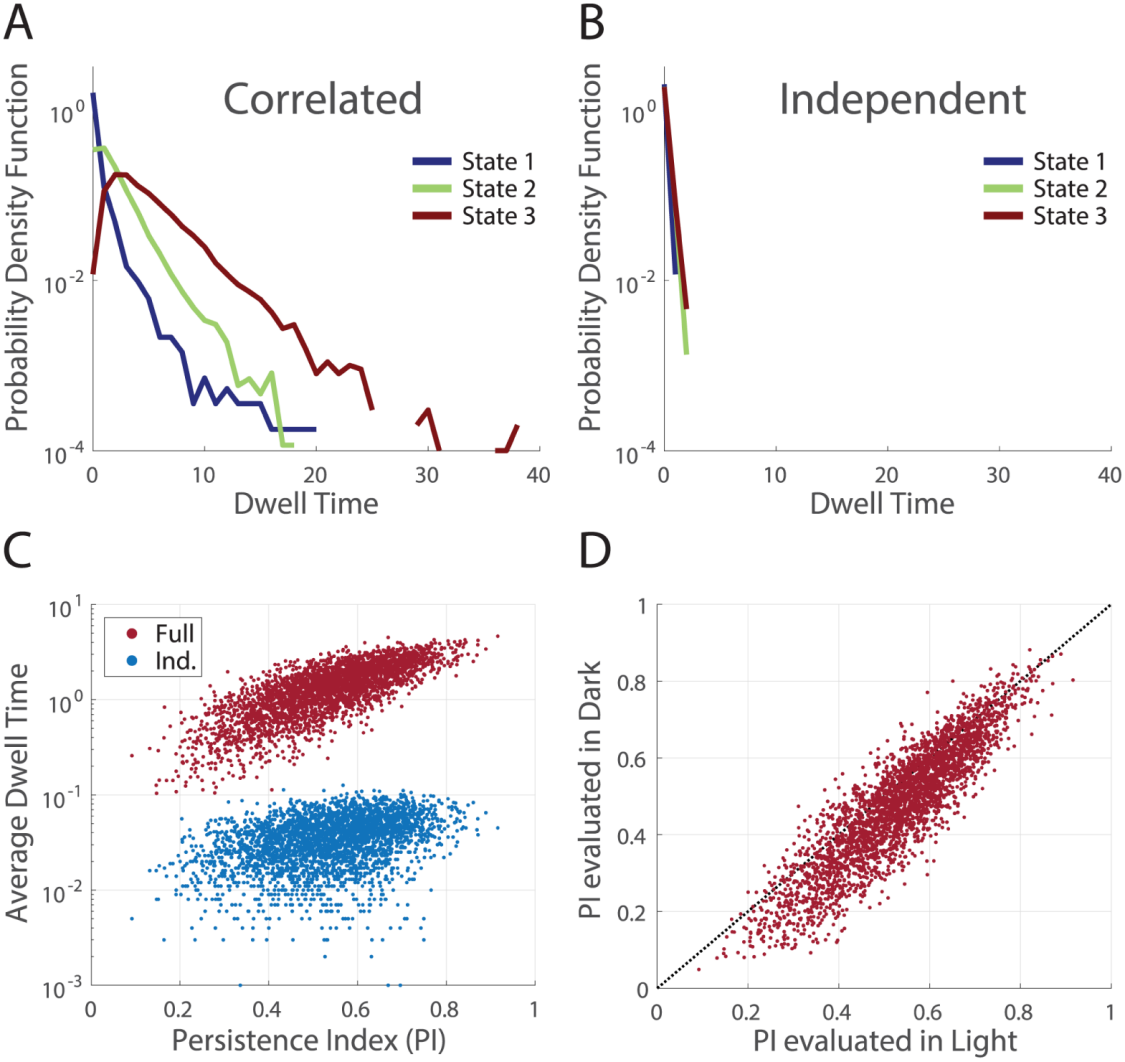
Visualizing the structure of the models through the dwell times in sampling. **A**. Distributions of dwell times for three sample states, estimated over 10^4^ separate instantiations of MC sampling from the full model. The persistence indices for states 1,2 and 3 were 0.092, 0.54, and 0.92, respectively. **B**. For the same states as in (**A**), distributions of dwell times estimated on shuffled (independent) data. **C**. Across the *N* = 3187 states with *K* = 12 spiking cells recorded in the data (M1, *light*) we measured the average dwell time (over 10^3^ MC runs) in the full (red) and independent (blue) models. These are plotted vs. the *PI* given by the full model. Note the logarithmic scale on the y-axis. **D**. The persistence indices for the same group of states are estimated using the maximum entropy model fitting the natural movie in the *light* (x-axis) and the *dark* (y-axis) adapted conditions.

We found significant differences in the distributions of dwell times across different initial states (Fig 9A). This demonstrated that Monte Carlo flow was trapped for a longer amount of time in the vicinity of particular states, consistent with subtle attractor-like properties in the geometric structure of the probability landscape. For the same initial states, average dwell times measured on the energy landscape for independent models were almost an order of magnitude shorter, indicating that these effects were due to the measured correlations (Fig 9B).

We searched for a measure that could capture the variability in dwell times across initial states in the full model, based on our intuition that a state that started near a local minimum would have a long persistence time before finite temperature MC sampling would move it far away. This led us to define a persistence index (*PI*) that captured the tendency of a state to remain near its starting point under MC sampling dynamics. Specifically, for a given state *R*, we define $PI=\hat{X}\left(R\right)\cdot \hat{R}$, namely the cosine of the angle between the initial state and the average next state. If the *PI* is close to 1, then the direction that the state *R* evolves towards under MC sampling is the state itself, and hence the state will remain the same.

Over all the initial states studied, we found a large positive correlation between the average dwell times and the persistence indices for the fully correlated population (correlation coefficient of 0.75, Fig 9C). This significant correlation justifies the use of the *PI* as a simpler proxy for the dwell time. In contrast, the correlation was only 0.38 when measured on the energy landscape of the independent model and dwell times were systematically smaller by orders of magnitude (Fig 9C blue).

The persistence index also allowed us to characterize the similarity of the neural code for the same natural movie between light and dark adapted conditions. If the structure in the energy landscape changed between the *dark* and *light* conditions, then one would expect the dwell times, and hence the persistence indices, to change as well. Instead, we found a strong correspondence across the *light* and *dark* experimental conditions, that was absent in the independent model (Fig 9D, S8 Fig correlation coefficient of 0.90 vs 0.37). To estimate the variability in this measure, we compared the *PI* across models inferred for two separate random halves of the *light* condition (S8 Fig, correlation coefficient of 0.97). Thus, the pattern of correlation measured in the two luminance conditions created similar structure in the system’s energy landscape, even though the detailed statistics of neural activity were quite different. This structure endows the population code with a form of invariance to light level that is not present at the level of individual ganglion cells.

## Discussion

Across different naturalistic stimulus conditions, we observed a similar sharpening of the specific heat as we increased the number of retinal ganglion cells, *N*, that were analyzed together. While this phase transition occurs at a temperature that does not correspond to any measured neural population, it nonetheless gives us insight into the collective properties of that real population. We’ve shown that aspects of this structure are conserved across large changes in average luminance, and that this structure is largely insensitive to minor perturbations of the correlation matrix. Our work ties together two seemingly disparate ideas: first, the observations of phase transitions in models of the real neural population [19, 21], and second, that the probability landscape might be organized into a discrete set of clusters [8, 62, 63]. We suggest that the former is a direct consequence of the latter.

### Clustering and glassy structure

Representing visual information with specific multineuronal activity states is an error-prone process, in the sense that responses of the retina to repeated presentations of identical stimuli evoke a set of activity states with different probabilities. A many-onto-one mapping of individual activity states to cluster identities naturally reduces this variability, thus endowing the population code with a form of error correction. In fact, this appealing and intuitive idea has been recently demonstrated for the retinal population code [62, 63].

Clustering of activity states manifested itself in the geometric structure of the probability distribution, which we characterized by several analyses, including Monte Carlo sampling dynamics, Monte Carlo flow vectors, and persistence indices. This structure was found to be preserved across variations in ambient luminance, and was robust to minor perturbations of the correlation matrix. This robustness was not obviously evident in the lower order statistics of the distribution (firing rates, correlations, spike count distribution), which were measured directly. For downstream readout mechanisms which access only the incoming retinal population code, such a robustness in the clustered organization constitutes a form of invariance in the retinal representation.

The variability in the interaction matrix gives rise to the variability that we observed in measures of persistence across states with the same number of spiking cells *K* (see Fig 9). In our picture in which the probability distribution over all neural activity states is organized into a set of clusters, some states are ‘attractor-like’. These states have a higher density of nearby states, which corresponds to lower energies, and thus traps states in their vicinity under MC sampling dynamics. Other states do not have this property at all, and hence the dwell time around these states under MC sampling dynamics approaches that of a network of independent neurons. This property depends crucially on the detailed structure of the pairwise interactions *J*_*ij*_. If for instance all the interaction matrix terms were set equal to a positive constant as in a ferromagnet, i.e. *J*_*ij*_ = *J*_0_, then the effective fields for all cells would have almost the same contribution from interactions, namely a quantity proportional to *KJ*_0_. The only variability in the conditional probabilities, *X*(*R*), that cells would experience would be due to the local fields and whether or not the cells were active in the state (which reduces the effective *K* by one); this variability would be overwhelmed with increasing *K*. As a result, the effective field would eventually tend to a constant for all cells at large enough *K*. However, we observe in our data that this is not true: the persistence index of states with the same *K* varied substantially (Fig 9).

The effect of variability in the distribution of interaction matrix terms has been studied extensively in spin glass models [56]. In order to convey some of our intuition about the low temperature regime, we will discuss a particular example of a glassy model and its relationship to our work. But keep in mind that while we believe there is a useful analogy between some of the properties of the glassy limit of the Sherrington-Kirkpatrick (SK) model [60] and our measured neural distribution, we are not claiming that our distributions are fit by the SK model.

The SK model is a model of all-to-all connectivity in a population, similar to the high degree of connectivity we observe in our inferred models of a correlated patch of ganglion cells. For example, cells have an average of 46 non-zero interactions per cell, out of *N* = 128 possible in the k-pairwise model in the *light* condition. The SK model itself has two regimes, characterized by the relationship between the variance ($\sigma_{J}^{2}$) and mean (*μ*_*J*_) of the interaction parameters, *J*_*ij*_. When the variability is large relative to the mean, the low temperature phase of the SK model is a spin glass phase, where the probability distribution over all activity states is characterized by an abundance of local maxima. The glassy SK model also undergoes a phase transition from a weakly-correlated paramagnetic phase to a structured spin glass phase as temperature is lowered. This transition is an ergodicity-breaking transition that is third-order (see below) [56, 64]. In other words, this transition is characterized by a significant reduction in the number of states available to the system, as the glassy state confines the system to particular valleys in the energy landscape.

A note is in order: ergodicity-breaking phase transitions can only be formally defined when there is no possibility that the system can escape a particular valley. This is only true mathematically in the thermodynamic limit (*N* → ∞), as a finite-sized system will always have a small but nonzero probability with which it can escape the phase space it is confined to. So we should always keep in mind that as we compare our data to the SK model, we need to consider the finite size limit of the SK model.

By comparison, when we scaled up the correlation strength *α* from values below the critical correlation strength, α*, to values above, we observed a phase transition where the smooth independent distribution wrinkled to form a set of attractor-like states in the energy landscape (Figs 7, 8 and 9), perhaps with the geometry of ‘ridges’ [65]. While this wrinkling transition does not strictly break ergodicity, like in the thermodynamic limit of the SK model, it does confine the system near attractor-like states. This confinement may in fact be quite similar to the ergodicity-breaking transition in the SK model. Indeed, our analysis of this transition suggests that it is consistent with a third-order transition, where there is a cusp in the specific heat at the critical temperature (i.e. a discontinuity in the derivative of the specific heat, not in its value; see S8 Fig). This behavior is reminiscent of the fact that the ergodicity-breaking transition for a spin glass in the SK model is also a third-order phase transition (see Figure 3 in ref. [60]).

These similarities suggest that the geometric properties of the probability landscape of the neural population are somewhat akin to the properties of the SK model in the spin glass phase, which is appealing for the connection between phase transitions and clustering of neural activity. But again, we are not arguing that our probability distributions over neural activity are exactly reproduced by the SK model. For instance, there are no local fields in the SK model, and these play a significant role in the properties of our maximum entropy models of neural data.

Fundamentally, error-correcting structure is not present in populations of independent neurons: if one neuron in an independent population misfires, that neuron’s information is lost [66]. The qualitative separability between the regimes of error-correction and independence is our proposed origin of the observed phase transition with respect to our temperature variable [19, 21].

### The phase diagram of the retinal population code

**Fig 10:**
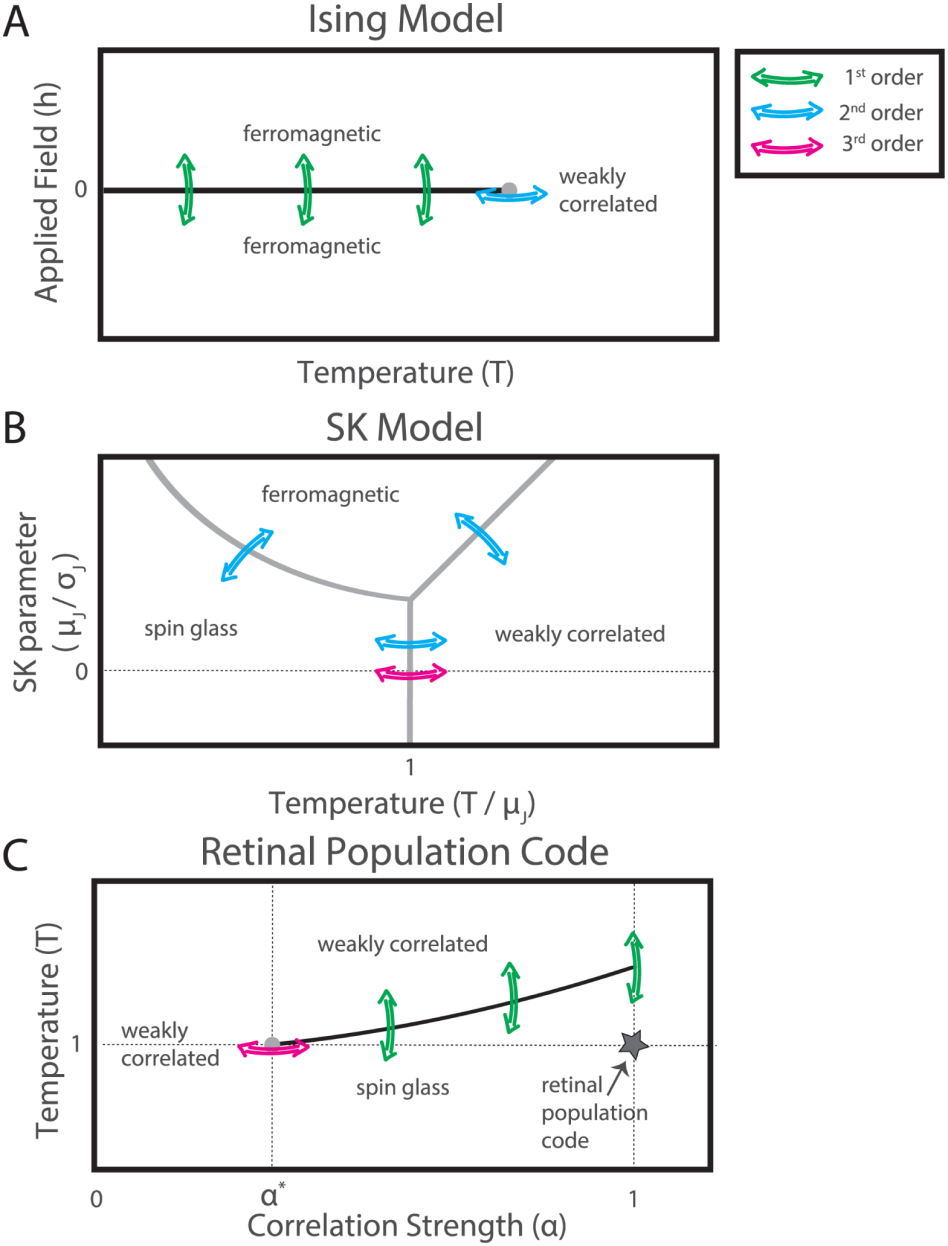
The phase space of the retinal population code. Sketches of the phase space of the nearest neighbor Ising ferromagnet (**A**), the Sherrington-Kirkpatrick model (**B**, following Fig.1 in Ref. [67]), and our work (**C**). Black lines indicate boundaries between phases which correspond to first-order phase transitions, while gray dots and gray lines correspond to transitions of higher-order (second- or third-order, depending on the model). Colored arrows indicate phase transitions of different orders. **A**. The line of first-order phase transitions is centered on an applied field *h* = 0, the critical point here is second-order. **B**. All transitions here are second-order, except in the spin glass limit, where they are third-order (the spin glass limit, *μ*_*J*_ ≪ *σ*_*J*_, is denoted by the dotted line). As explained later on in the text, the second-order transitions here are marked by a discontinuity, not divergence, in the specific heat. **C**. In our work, a third-order phase transition as a function of correlation (at *α* = α*, *T* = 0) is the origin of a line of first-order phase transitions as a function of temperature. The location of the real neural population code is denoted by a star.

To summarize these ideas, we present a suggested picture of the phase transitions studied in our work, by comparison with the Ising ferromagnet and the SK spin glass models (Fig 10). In the Ising ferromagnet at low temperature (Fig 10A), the constant and equal interactions cause all the cells to tend to be active or quiet simultaneously. At high temperature, fluctuations wash out the interactions and the system is in a weakly correlated (paramagnetic) phase. As temperature is decreased at zero applied field, the system reaches a critical point where it chooses the all-active or all-quiet half of phase space (grey dot in Fig 10A). Below the critical temperature the two halves of phase space are separated by a line of first-order phase transitions that separates a ferromagnetic phase with all the spin aligned in one direction from a similar ferromagnetic phase with all the spins aligned in the opposite direction (black line in Fig 10A).

As described above, the SK model has both a spin glass and a ferromagnetic limit (Fig 10B, sketched following [67]). In the SK model in the spin glass limit (*μ*_*J*_ ≪ *σ*_*J*_), decreasing the temperature at zero applied field causes the model to freeze into a spin glass phase. This ergodicity-breaking phase transition is third-order (Fig 10B, magenta arrows).

In our picture of the retinal population code, a third-order phase transition occurs when the correlation strength *α* is increased from 0 at *T* = 1 (gray dot and magenta arrow in Fig 10C). In this phase transition, the distribution of neural activity wrinkles to form a set of ridges in the probability landscape. Because firing rates are constrained during this manipulation, this manipulation is analogous to varying temperature at zero applied field in the nearest neighbor Ising ferromagnet, and to varying temperature in the spin glass limit of the SK model. Increasing the temperature in our models of the retinal population when the correlation *α* is greater than α* causes the distribution to melt to a weakly correlated state in a transition that is first-order (see Fig 7B). Because of the local fields in our models, variations in temperature are accompanied by changes in firing rate, and this is most similar to varying the applied field, *h*, in the Ising ferromagnet. The analogy between the axes in the Ising ferromagnet and our model only extends to the horizontal (temperature) axis of the SK model: here the phase space far from the dotted line is shown for clarity.

So in summary, the main phase transition that we see when we change temperature is first-order, as is the transition between ferromagnet states in the Ising model when the applied field is changed. In both cases, there is a substantial change in the state of individual elements—the firing rate of neurons in the retinal population and the magnetization of spins in the Ising model. Furthermore, the 3rd-order phase transition that we observe when *α* increases above α* is reminiscent of the 3rd-order phase transition in the SK model as a function of temperature in the limit of highly variable interactions. In our case, increasing *α* increases the strength of correlations, while in the SK model, lowering temperature increases the impact of interactions on the network state. This analogy provides support for the conclusion that the low temperature phase of the retinal population resembles a spin glass.

### First- versus second-order phase transitions

In statistical physics, the peak in the specific heat that we observed in our models (Fig 3) could be consistent with two types of phase transition, which are classified by the order of the derivative of the free energy which exhibits a discontinuity. In statistical mechanics, every physical system can be described on a macroscopic level by a free energy function. When the system transitions between phases, some order of the derivative of this free energy function will have a discontinuity. The first-order derivative of free energy versus temperature is the entropy, so when there is a discontinuity in the entropy, the system is said to exhibit a ‘first-order’ phase transition. The second-order derivative of free energy versus temperature is proportional to the specific heat, so a system with a discontinuity in the heat capacity exhibits a ‘second-order’ phase transition.

The ambiguity we suffer in interpreting our data arises due to the fact that phases and transitions are rigorously defined in the thermodynamic limit which is when the system size *N* is taken to infinity. At finite sizes, it may be difficult to tell these two possibilities apart. The first possibility is that the observed phase transitions exhibit a divergence in the specific heat, making them second-order. This would indicate criticality in the retinal population code [19, 21, 27]. The second possibility is that the entropy is discontinous and that the specific heat exhibits an infinite value only at the critical temperature (i.e. the specific heat has a delta-function form). This would be a first-order transition, and it does not indicate criticality. Both of these hypotheses would be consistent with a sharpening of the specific heat as the system size increased (Fig 3). How could we distinguish definitively between these two types of transition and what are the consequences of the difference?

This issue would be resolved if we could convincingly relate the properties of our model to some well known example in physics that does have a critical point. There are two examples that we have in mind here. The first is the symmetry-breaking mechanism for criticality (see for example chapter 142 onwards in [5]). For concreteness we’ll take the example of the structure in the nearest neighbor Ising ferromagnet, where all the non zero interactions *J*_*ij*_ are a positive constant and there are no other parameters. In the high temperature state, the system has the ability to visit any one of the 2^*N*^ possible states, and the average firing probability for each neuron is 0.5 in a single time bin. In the low temperature state, the interactions cause all the cells to behave similarly, either mostly silent or mostly active, with some fluctuations allowed by the temperature. Importantly, however, the two phase spaces centered on all-silent and all-active are separated by an energy barrier that increases with system size. Because of this property, at large enough system sizes the available phase space in the low temperature state is reduced by a factor of two. The low and high temperature phases are known as low and high symmetry states, respectively, and it is the change in symmetry at the phase transition which leads to discontinuities in the specific heat. The critical point here is thus characterized by a reduction in symmetry where the symmetry described in this example is the deviation of the average firing rate from one half.

In our models, the presence of local fields means that all cells have some bias towards silence or activity. As a consequence, the symmetry in firing rate is absent regardless of the temperature, and no symmetry breaking can occur with respect to changes in firing rates. There might be some other symmetry that is broken at the transition between phases, but no one has identified it yet [19, 21]. Failing to identify a symmetry breaking mechanism in our analysis does not prove that the peak in the heat capacity is a first-order phase transition, but it is consistent with this interpretation.

The second type of critical point that we’ve considered is the type that occurs in a spin glass, as in the SK model. These critical points are also characterized by a reduction in the size of the available state space, as in the symmetry breaking example described above. Because these transitions occur between paramagnetic and glassy regimes, they are also candidates for describing the transition that we observe in Fig 3. However, these critical points in glassy systems are not, to our knowledge, characterized by a divergence in the specific heat. The second-order transition in the SK model is discontinuous in the specific heat, but not divergent (see Figure 3 in ref. [60]). If such a critical transition did occur, it would not lead to a sharpening of the specific heat as we observe in Fig 3. Instead, it would resemble the transition we observe as a function of increasing correlation strength, *α*, where the specific heat is either discontinuous or has a cusp at α* (Fig 7 and see our discussion in the supplement, S8 Fig). Additionally, such critical points are typically marked by a divergence in some higher order statistic, such as the nonlinear susceptibility. Our measurements of the nonlinear susceptibility have not shown such a result (S11 Fig).

To summarize, we have considered two natural mechanisms that could connect our observed peak in the heat capacity versus temperature to a well known critical point in statistical physics, and we find that these mechanisms are simply not consistent with the observed properties of the data. It is possible that some other analogy provides a connection between criticality and our observations, but we are not aware of it. So, taken together, we believe that these observations argue against the hypothesis that the retinal ganglion cell population is poised at a critical point.

### Implications of first- versus second-order phase transitions

Criticality implies that there is something special in the distribution of neural responses: for example, that the specific heat is maximized with respect to some properties of the retinal circuit, and hence that “the distribution of equivalent fluctuating fields must be tuned, rather than merely having sufficiently large fluctuations” [19]. Our analysis in which we scaled the strength of all pairwise correlations by a constant factor is not consistent with the notion of fine tuning. Specifically, we could decrease pairwise correlations by a factor of more than 2 without significant changes in the specific heat (Fig 7C) or the geometric structure of the probability distribution (Fig 8). Additionally, we actually observed higher peaks in the specific heat at *T* ≠ 1 in the partially correlated networks than in the fully correlated networks (Fig 7). This fact also argues against the idea that the heat capacity of the system is strictly maximized by some principle requiring fine tuning.

**Fig 11:**
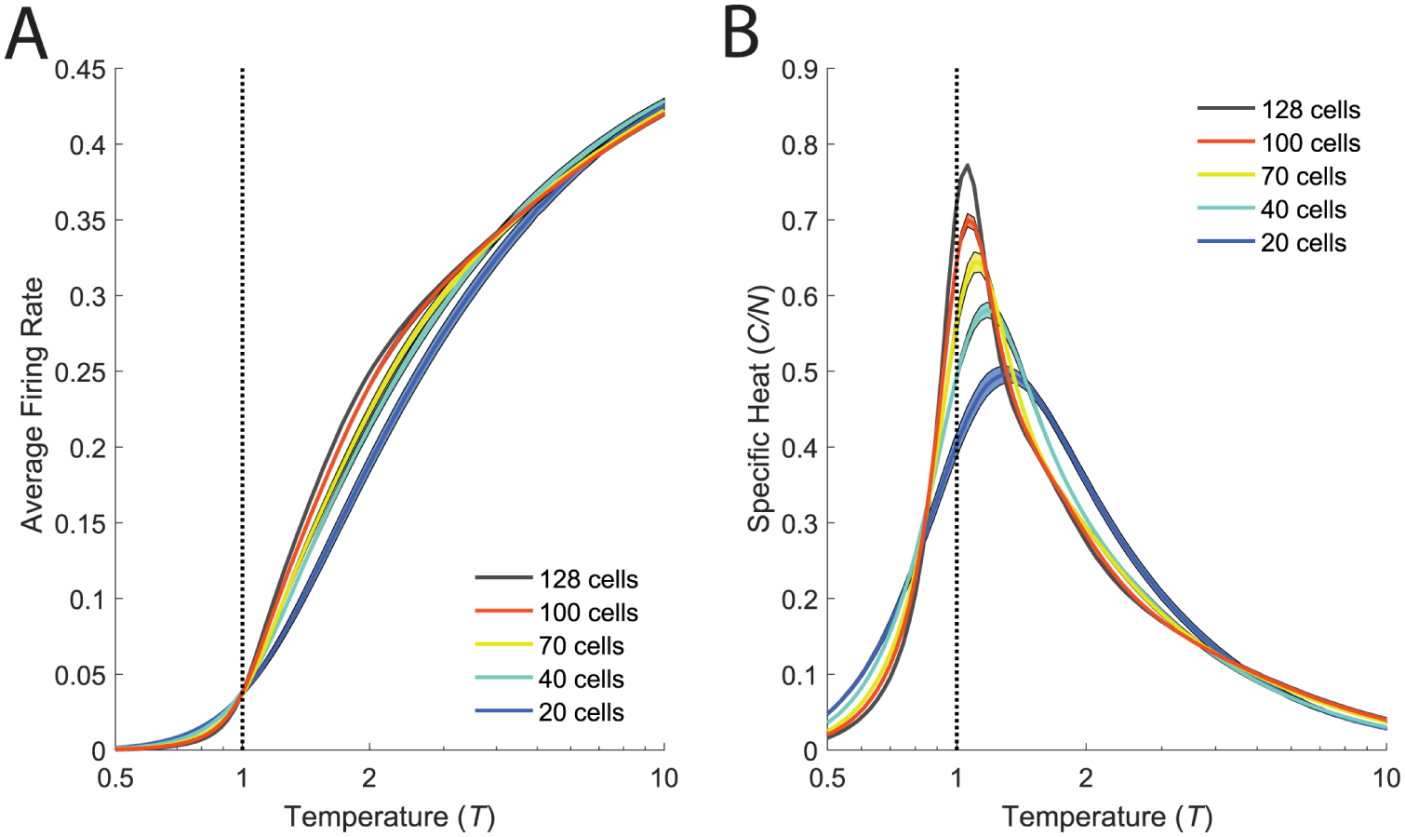
A sharp increase in firing rates accompanies the phase transition. **A.** Firing rates averaged over cells plotted as a function of the temperature, *T*, during the same annealing procedure used to estimate the specific heat (**B**, reproduced from Fig 3 for convenience). Model parameters were inferred for the k-pairwise maximum entropy model, in the *light* condition. Error bars (shaded regions) are the standard error of the mean.

The interpretation of the peak in the heat capacity as a first-order phase transition also provides an explanation for the proximity of the system to the transition. Our argument is that correlations in the distribution create a phase which is qualitatively different than the high temperature phase. This qualitative difference then requires that there be a a transition between these two regimes. Furthermore, the sparseness of neural activity implies that the zero-temperature limit of the model is the all-silent state. Consequently, the average firing rates must follow a sigmoidal function of temperature, starting at zero for *T* = 0, rising for *T* > 0, and then saturating at 0.5 firing probability for *T* → ∞. This sigmoid sharpens into a step with increasing system size, with the width of the step corresponding to the area over which finite-size effects smear out the phase transition (see Fig 11). Such a step-like change in the firing rate is most consistent with a first-order phase transition (where first-order derivatives of the free energy, such as the firing rates, change discontinuously). Given this context, constraining the average firing rate to be some small but nonzero value forces the system to be poised in the vicinity of the phase transition. Thus, we believe that the proximity of the system to the transition point simply follows as a consequence of constraining the structured phase to have neurons with low firing rates.

In addition, it is difficult to reconcile the notion that the retinal code is poised at a very special, fine-tuned operating point with the observation that similar peaks in the specific heat arise in many circumstances, including very simple models [27]. In this sense, we agree with the conclusion that Nonenmacher and colleagues reached that this notion of fine tuning is not well supported by these wider considerations. In contrast, a first-order phase transition does not imply that something is “special” or “optimal” about the retinal population code. Instead, the hypothesis that the population is in a low temperature state is appealing, because this state can be robustly present without fine tuning.

To sum up, our heat capacity analyses do not clearly disprove the hypothesis that the system is poised at a critical point. However, first-order phase transitions are far more common in nature than second-order transitions. They do not require any special tuning of the parameters of the system. Thus, the interpretation that the neural population is in a low temperature state serves as a simple hypothesis that is consistent with all of our data. This interpretation has additional value, because it suggests a connection between the phase transition and the emergence of structure in the probability landscape. And in fact, our analyses have directly confirmed this connection (Figs 8 and 9).

### Relationship to other studies

While we believe there are no inconsistencies in our empirical findings and those of other studies that focused on criticality in retinal population codes, our interpretations differ substantially [24, 25, 27].

A recent study tested the generality of phase transitions in simulations of retinal ganglion cells [27], simulating networks of neurons in the retina, and then estimating the specific heat following procedures similar to those presented here. Their results are consistent with ours in that phase transitions were robustly present in different networks of neurons, and the presence of these phase transitions was largely invariant to experimental details. Similar to us, they also found that the sharpness of the peak in the specific heat was systematically enhanced by stronger pairwise correlations. Because Nonnenmacher et al. also found this behavior in very simple models, such as homogeneous neural populations, they concluded that the sharpening peak in the heat capacity does not necessarily provide insight, by itself, into the structure of the population code. We agree. However, because we went on to analyze the structure of the probability landscape for our real, measured neural populations, we could show that the emergence of the peak was related to the clustering of neural activity patterns (Figs 8 and 9). The relationship between the low temperature phase and clustering is not understood in general. Studies of homogeneous models of neural populations demonstrate that the low temperature phase is not sufficient for clustering [27], while the current study suggests that the low temperature phase might be necessary. Other factors, such a sufficient heterogeneity of single neuron properties, are presumably also required. In any case, we interpret the robustness of a phase transition in correlated neural populations not as an argument that this property is trivial, but instead as evidence for the generality of clustering in neural population codes.

A separate line of work has investigated the presence of Zipf-like relationships in the probability distribution of neural codewords [23–25]. A true Zipf Law is intimately related to a peak in the heat capacity at *T* = 1, and Schwab et al. found that a Zipf Law was present under fairly generic circumstances, in which neural activity was determined by a latent variable (e.g., an external stimulus) with a wide range of values [24]. Again, this result is broadly consistent with our finding of great robustness of the low temperature state, and we interpret this a positive evidence for these properties being generically present in correlated neural populations. However, we can’t make more detailed comparisons to [24, 25], because we have not chosen to analyze Zipf-like relationships in our experimental data. There are several reasons for this choice: 1) we can only sample ∼2 orders of magnitude in rank (S9 Fig), making it difficult to estimate power law exponents; 2) we typically observe small deviations from the power law trend, and we are uncertain about how to interpret the importance of these “bumps”.

In our study, we have characterized the neural response in a single time bin, ignoring the role of temporal correlations across time bins. One can extend our approach to include temporal correlations by concatenating multiple time bins into each neural codeword. When the number of total time bins was systematically increased in such a manner, the peak in the specific heat sharpened substantially [21]. The authors interpreted these results as further evidence in favor of the critical properties of neural population codes. However, in all cases in both our study and of [21], the peak was above *T* = 1, consistent with our interpretation that neural populations are in a low temperature state. Since increasing the number of time bins this way drastically increases the complexity of the distribution, this treatment of temporal correlations increases the structure of the low temperature state in a manner similar to an increase in the number of neurons analyzed together, *N*.

Using several different analysis methods, neural activity evoked by repeated presentations of the same stimulus has been shown to form clusters in the space of all possible activity patterns. Zero temperature descent in the energy landscape defined by the maximum entropy model mapped a large fraction of all neural activity patterns to non-silent energy basins, which were robustly activated by the same visual stimulus [16, 68]. Mapping neural activity patterns to latent variables inferred for a hidden Markov model revealed similar robust activation by the stimulus [63, 65]. Huang et al. found a form of first-order phase transition as a function of the strength of an applied external field, from which they concluded that the energy landscape formed natural clusters of neural activity with no applied field [69]. Ganmor et al. recently uncovered a striking block diagonal organization in the matrix of semantic similarities between neural population codewords [62], arguing for a clustered organization of neural codewords. All these analyses are likely to be different ways to view the same underlying phenomenon, although a detailed exploration of the correspondences among these methods is a subject for future work.

### Connection to neural codes in other brain regions

Are our results specific to the retina? In our approach, the collective state of the retinal population code is entirely determined by the pattern and strength of measured correlation. There is nothing about this pattern of correlation that makes specific reference to the retina. This means that any neural population having similar firing rates and pairwise correlations would also be in a similar collective state. Additionally, the strength of pairwise correlations we report here is smaller than or comparable to those reported in higher order brain areas, such as V1 and MT [57, 58, 70, 71]. This suggests that the collective state of neural activity, which arises due to a clustering of neural activity patterns, could occur throughout higher-order brain regions in population recordings of a suitable size (*N* >100).

## Materials and methods

### Ethics statement

This study was performed in strict accordance with the recommendations in the Guide for the Care and Use of Laboratory Animals of the National Institutes of Health. The protocol was approved by the Institutional Animal Care and Use Committee (IACUC) of Princeton University (Protocol 1828).

### Experimental recordings

We recorded from larval tiger salamander (*Ambystoma tigrinum*) retina using the dense (30 *μ*m spacing) 252-electrode array described in [1]. In Experiment #1, which probed the adaptational state of the retina at normal and low ambient illumintation levels, the salamander was kept in dim light and the retina was dissected out with the pigment epithelium intact, to help the retina recover post dissection and adjust to the low ambient light levels in the *dark* condition. The rest of the procedure in Experiment #1, and the full procedure for Experiment #2, followed [1].

### Stimuli

The chromatic checkerboard stimulus (CC) consisted of a random binary sequence per color (R,G,B) per checker, allowing 8 unique values for any given checker. Checkers were 66 *μ*m in size, and refreshed at 30 Hz. There were two (gray scale, 8 bit depth) natural movies used: grass stalks swaying in a breeze (M1, 410 seconds) and ripples on the water surface near a dam (M2, 360 seconds). Both were gamma corrected for the CRT, and displayed at 400 by 400 pixel (5.5 *μ*m per pixel) resolution, at 60 Hz.

### Experimental details

In Experiment #1, after adapting the retina to the absolute dark for 20 minutes, we recorded in the *dark* condition first (by placing an absorptive neutral density filter of optical density 3 [Edmund Optics] in the light path), stimulating with (CC) for 60 minutes, and with (M1) for 90 minutes. The filter was then switched out for the *light* condition, in which we recorded for an additional 60 minutes of (M1) and another 60 minutes of (CC). To avoid transient light adaptation effects we removed the first 5 minutes of each recording (10 minutes from the first checkerboard) from our analysis. During stimulation with (M1) we sampled 340 sec long segments from (M1) with start times drawn from a uniform distribution in the [0 60] second interval of (M1). The spike sorting algorithm [1] was run independently on the recordings in response to (M1) at the two light levels, generating separate sets of cell templates at the two light levels, which were then matched across the two conditions, yielding *N* = 128 ganglion cells. The spike trains were then binned in 20 ms time bins and binarized, giving 2.5 ⋅ 10^5^ states in the *dark* and 1.75 ⋅ 10^5^ states in the *light*. For the recordings from the checkerboard (in both light conditions), the templates from the *light* recording were used to fit the electrode activity. Across all four stimulus conditions this left us with *N* = 111 cells for the comparisons of natural movies to checkerboard. The checkerboard in the *light* condition was binned into *N* = 145623 states.

In Experiment #2, we alternated stimulation between (M1) and (M2) every 30 seconds, sampling 10 sec segments from both movies. For our analysis here we worked with the statistics of the last 9.5 seconds of each 30 second bout, yielding 8 ⋅ 10^4^ states per stimulus condition, for *N* = 140 cells.

### Maximum entropy model inference

Our maximum entropy model inference process implements a modified form of sequential coordinate gradient descent, described in [16, 47, 48], which uses an L1 regularization cost on parameters. For the k-pairwise model, we inferred without a regularization cost on the local fields. Further details are given in the Supplement (S1 Text)

To measure the heat capacity we simulated an annealing process. Initializing at high temperatures, we monte carlo sampled half a million states per temperature level (in 100 parallel runs of 5 ⋅ 10^3^ samples each), initializing subsequent lower temperature runs with the final states of preceeding higher temperature runs. The heat capacity at a particular temperature was then evaluated as *C* = (〈*E*^2^〉 − 〈*E*〉^2^)/*T*^2^.

### Network of LN neurons

Our model LN neurons were estimated over the chromatic checkerboard recording in the *light* condition. For each cell, the three color-dependent linear filters (the full STA) were weighted equally before convolution with the stimulus for an estimate of the linear response *q*. The non-linearity was estimated over the same data by Bayes rule, *P*(spike|*q*) = *P*(*q*|spike)*P*(spike)/*P*(*q*). Spike trains were simulated from a novel pseudorandom sequence put through the model’s filters and non-linearity, with the non-linearity shifted horizontally to constrain the firing rates of the neurons to be the same as in the experimental recordings. The result was binned and binarized to yield *N* = 145623 states, for which we inferred the k-pairwise model.

## Supporting information

S1 Fig Comparing error estimates. Two different estimates of error, the standard error of the mean and standard deviations over bootstrap resamples yield similar estimates of error.
(EPS)

S2 Fig Quality of fitting, light dataset. The high accuracy with which the k-pairwise maximum entropy models reproduce the firing rates, pairwise correlations, and spike count distribution in the real data. Additionally, the convergence of the algorithm.
(EPS)

S3 Fig Quality of fitting, dark dataset. Same as S2 Fig, except for the *dark* dataset.
(EPS)

S4 Fig Matching other statistics in the distribution, light dataset. How well average triplet correlations, conditional probabilities of a spike, and the probabilities of states are captured by the k-pairwise maximum entropy model fit to the data in the *light* dataset.
(EPS)

S5 Fig Matching other statistics in the distribution, dark dataset. Same as S4 Fig, except for the *dark* dataset.
(EPS)

S6 Fig Comparing analytical solutions to shuffled-data model fits. (EPS)

S7 Fig Pairwise maximum entropy model results for natural movies. These are qualitatively similar to the results presented in the main text, where we work with the additional constraint on spike count.
(EPS)

S8 Fig Characterizing the transition with respect to correlation strength *α*. We elaborate on our analysis and interpretation of the dependence of the specific heat on correlation strength *α* system size *N*.
(EPS)

S9 Fig Persistence indices. A little more detail on the persistence index.
(EPS)

S10 Fig Zipf relationships. (TIF)

S11 Fig Nonlinear susceptibility. A calculation that is consistent with a first-order intepreration for the temperature-driven phase transition.
(EPS)

S1 Text Detailed description of the supplementary files. (PDF)
