# Supplementary figures and images for: The structured ‘low temperature’ phase of the retinal population code
Source: PLoS Comput Biol. 2017 Oct 11;13(10):e1005792. doi: 10.1371/journal.pcbi.1005792 (PMC5654267; doi:10.1371/journal.pcbi.1005792)

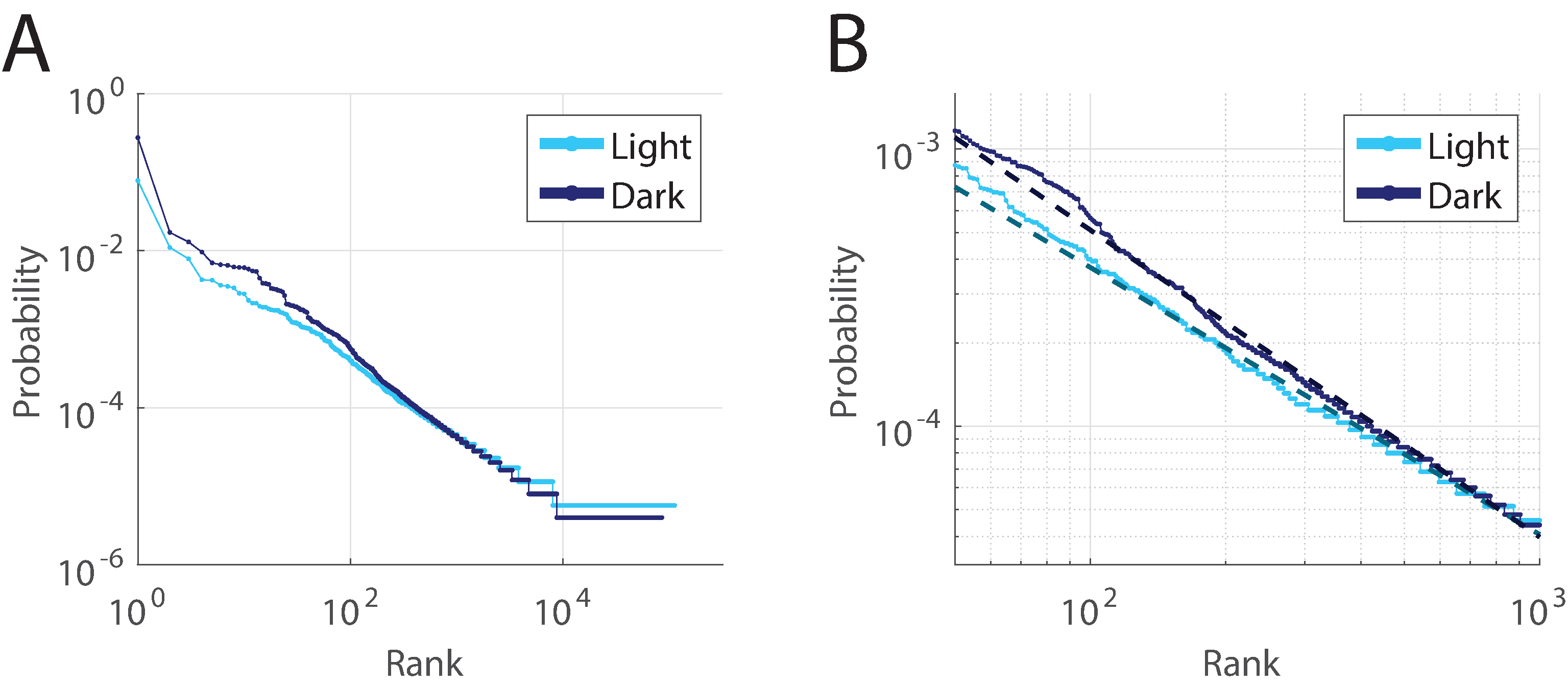

Supplement: S10 Fig — (TIF) [file pcbi.1005792.s010.tif]
