# Supplementary material for: The structured ‘low temperature’ phase of the retinal population code
Source: PLoS Comput Biol. 2017 Oct 11;13(10):e1005792. doi: 10.1371/journal.pcbi.1005792 (PMC5654267; doi:10.1371/journal.pcbi.1005792)
Supplement: S1 Text — (PDF) [file pcbi.1005792.s012.pdf]

Contents

Estimating Chromatic Sensitivities 2

Model Inference and Fitting the Expectation Values 2

Matching Other Statistics In the Distributions of Neural Responses 5

Analytical vs. Model Fits for Independent Networks 8

Pairwise Maximum Entropy Model Results 11

Characterizing the Transition With Respect to Correlation, α 12

Persistence Indices 13

Zipf-like Relationships in the Distribution of Codewords 16

Nonlinear Susceptibility 17

References 19

Estimating Chromatic Sensitivities

We estimated color-dependent spike triggered averages (STAs) for each ganglion cell. To identify the Receptive Field (RF) centers within each STA, we took the normalized dot product of each checker's time course with the time course of the extremal checker. Checkers were identified as belonging to the RF center if this dot product exceeded 0.3, and they were connected by 4-neighbor connectivity to the extremal checker. The time course of the sum over the RF centers was defined as the temporal time course of the STA.

To estimate each ganglion cell's chromatic sensitivity we found (across all three color-dependent temporal time courses) the absolute maximum deviation from zero. We then took the three values of the color dependent STA's at this time point, and normalized, to generate a 3 x 1 unit vector $\Phi_i^{(d/l)}$ representing the color sensitivity of each ganglion cell i in each light condition (d/l). To model the chromatic sensitivity of the 'red rod' or 'green cone' photopigment in our experimental setup, we measured the spectral output of the monitor's red, green and blue guns directly [Ocean Optics USB 2000]. We took estimates of the red rod and green cone absorption spectrum from the literature [Perry et al., 1991; Baylor et al., 1987; Cornwall et al., 1984], and took the dot product of these spectra with the measured monitor outputs. Our estimates of the green cone and red rod photopigment sensitivites to the red, green and blue monitor guns were $X_g = [1.15,\ 1.23,\ 0.42]$ and $X_r = [0.20,\ 1.49,\ 0.86]$. For example, for cell i in the *dark* adapted condition (d) the projection of the STA onto the rod (r) was defined as $z_{i,r}^d = X_r \cdot \Phi_i^d/3$. The rod-cone separation for each projection was defined as the distance of the projection to the unity line (Fig. 1B in the main text) normalized by the distance of the rod-cone overlap (red square, Fig. 1B in the main text) to the unity line.

Model Inference and Fitting the Expectation Values

We binned each ganglion cell's spike train in 20 ms time windows, assigning 0 for no spikes and 1 for one or more spikes, $r_i = [0, 1]$. We denote a particular population activity pattern as $R = \{r_i\}$. Maximum entropy models describing the distributions of time-averaged variables are fit with

unitless 'energies':

$$E_{\mathrm{pairwise}}(R) = \sum_i^N h_i r_i + \sum_{i,j\neq i}^N J_{ij} r_i r_j \tag{1}$$

$$E_{\mathrm{k}-pairwise}(R) = \sum_i^N h_i r_i + \sum_{i,j\neq i}^N J_{ij} r_i r_j + \sum_{k=1}^K \delta\left(\sum_i r_i - k\right)\lambda_k \tag{2}$$

With δ the Kronecker delta. This model is constrained to match the expectation values $\langle r_i \rangle$, $\langle r_i r_j \rangle$ and $\langle \sum_i r_i \rangle$, averaged over the training data.

The maximum entropy model inference process implements sequential coordinate gradient descent, described in [Dudik et al., 2004; Broderick et al., 2007; Tkačik et al., 2014] , which uses an L1 regularization cost on parameters, derived from the expected measurement error on the corresponding expectation values. For the k-pairwise model, we inferred without a regularization cost on the local fields. This emphasises solutions to the inference of an 'independent' nature, so that terms in the interaction matrix, and the k-potential, are inferred only when necessary.

During inference, we randomly selected four fifths of the data states for training, and withheld the rest for testing. All models presented in our work were inferred with the same preset hyperparameters:

Round #	1	2	3	4	5	6
# of Iterations	200	200	200	300	300	800
# of Hist MC Resamples	7	5	3	1	0	0
# of MC Samples (x 10^6)	0.2	0.4	0.65	0.8	0.9	1

For example, Round 2 was preset to 200 iterations. On each iteration, $4 \cdot 10^5$ monte carlo samples were drawn to estimate the expectation values. These same samples were re-used with histogram MC resampling [Broderick et al., 2007] 5 additional times. We converged to these choices of hyperparameters after finding that they were sufficient for the two main datasets.

On each round we also estimated the rms of all mistakes in expectation values (normalized by the

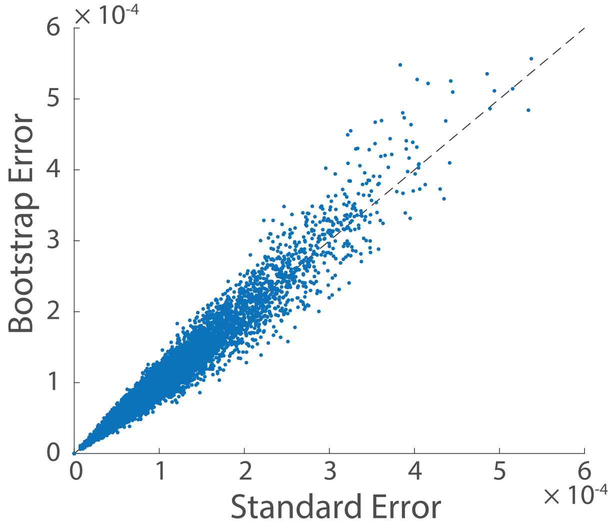

Figure S1: Comparing Error Estimates. Error estimate for the pairwise expectation value $\langle r_i r_j \rangle$ in the *light* dataset, for each pair of cells, over $128 \cdot 127/2$ pairs. Bootstrap resamples were estimated as standard deviations over 30 resamples of the data. The standard error was given by the standard deviation over samples, divided by the square root of the number of samples ($1.4 \cdot 10^5$ training data states). The dashed line is the unity line.

appropriate error estimate, i.e. these were z-scores) that we were fitting, excluding those rare expectation values which were zero in the training data. If the rms of z-scores fell below 1 on a particular round, that round exited early, and we progressed to the next set of hyperparameters. These early exits were implemented to avoid overfitting in the networks with artifically low correlation (such as the $\alpha = 0.1$ network in the scaled covariance manipulation in the main text).

The error estimate in the z-scores had contributions from both error estimates in the testing data and MC samples drawn from the model. We used the standard error of the mean as estimates of the error, because bootstrap resampling to estimate the errors of our expectation estimates was not always an available option. For example, in the manipulation where we scaled the covariance matrix by a constant α, we had direct access to the new covariance matrix, but there were no 'data' states to resample. Thus for consistency across all inference procedures we worked with the standard error of the mean. We have found the two estimates of variability to be comparable in our data (Fig. S1).

We found that fit quality improvement (estimated on the testing data) slowed substantially after the first several thousand parameter updates [Fig. S2 E,G and Fig. S3 E,G]. Note that the error bar

depends on the number of samples in both the data (there was 4X more training data than testing data), and in the model (the number of MC samples on a given iteration). Thus on each subsequent increase in MC sample number the estimated errors decreased and the z-scores increased.

In detail, we show the quality of fits for the two datasets. These are from the recording in Experiment #1, for the natural movie stimulus (M1), in the two light conditions (Fig. S2 and Fig. S3, panels A-G).

Matching Other Statistics In the Distributions of Neural Responses

To test how well our models captured aspects of the distribution that were not explicitly fit by the models, we followed two recent publications [Tkačik et al., 2014; Ganmor et al., 2011].

We estimated the triplet correlations $C_{ijk} = \langle (r_i - \langle r_i \rangle)(r_j - \langle r_j \rangle)(r_k - \langle r_k \rangle) \rangle$ over all states in the data, and over $5 \cdot 10^5$ MC samples drawn from the model. These were then sorted by value in the data, and grouped into one thousand equally populated groupings. The mean and standard deviation over those groupings were close to the unity line (Panel A in Fig. S4,5). These results appear very similar to those in [Tkačik et al., 2014] (there is a factor of 8 shift in the absolute values of C_{ijk} arising from a change in variables from their spin notation, [-1,+1], to our spiking notation, [0,1]).

Maximum entropy models also do fairly well in matching the conditional probability of a spike [Tkačik et al., 2014]. Specifically, for any given state R, each cell i has a predicted probability of a spike:

$$x_i(R) = \exp(h_i^{\text{eff}}(R))/(\exp(h_i^{\text{eff}}(R)) + 1) \tag{3}$$

$$h_i^{\text{eff}}(R) = h_i + 2 \sum_{j \neq i} J_{ij} r_j + \lambda_{K+1} - \lambda_K \tag{4}$$

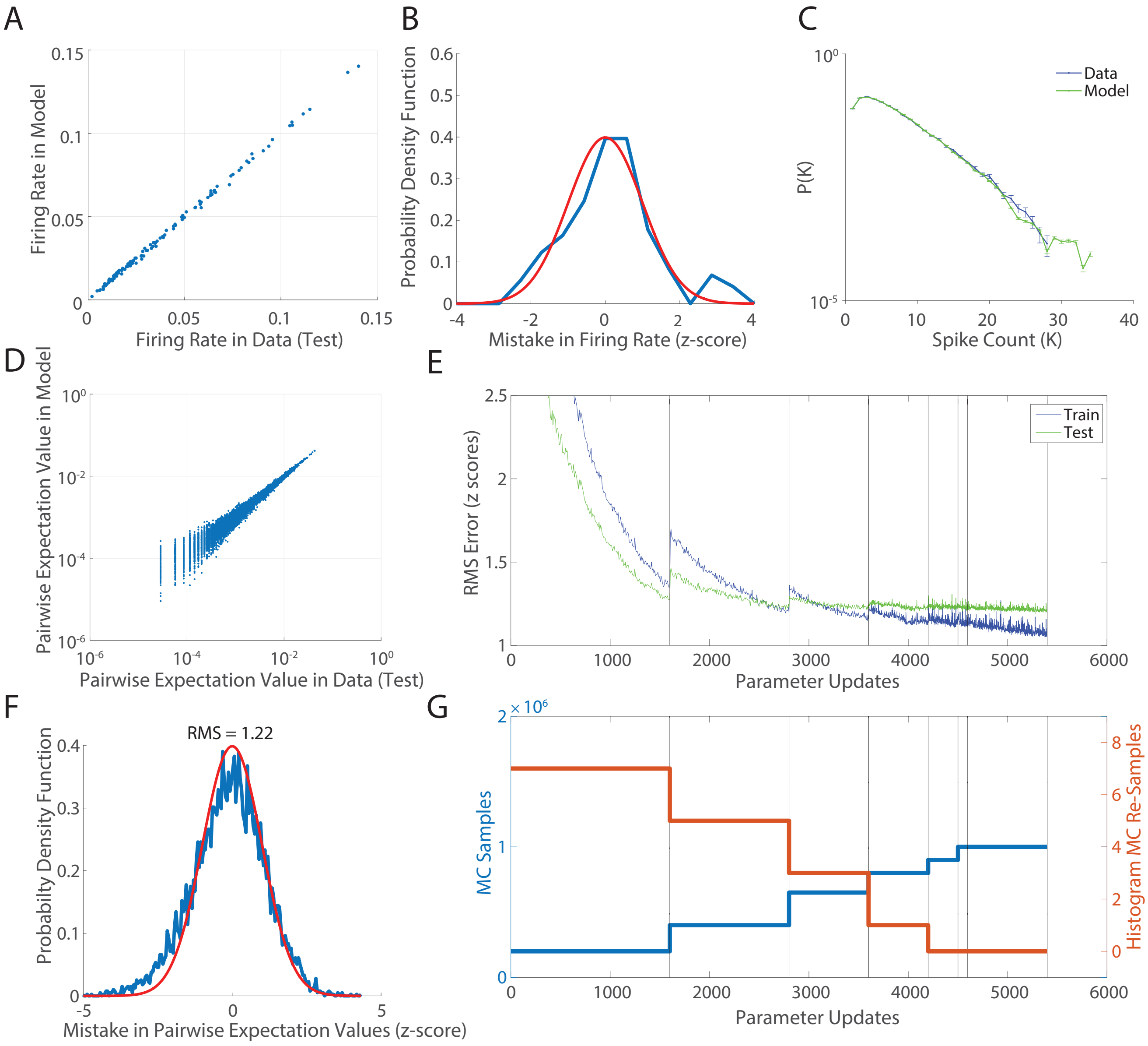

Figure S2: Quality of Fitting. Experiment #1, M1, Light. Dataset of $N = 128$ ganglion cells recorded over a total of $1.75 \cdot 10^5$ states. The estimates of statistics in the data here use withheld (testing) data. **A**. Matching the firing rates estimated in the data with the model. These are expectation values over the binarized 20ms bins. Error bars are not shown, see panel (**B**). **B**. Distributions of the z-scores of mistakes in estimating the firing rates in panel **A**, with a numerical gaussian in red. **C**. Matching $\mathrm{P}(K)$ in the model (green) to that estimated from the data (blue). Error bars are standard error of the mean. **D**. Pairwise expectation values ($\langle r_i r_j \rangle$) estimated from the model (y-axis) plotted against the pairwise expectation values measured experimentally (x-axis), for all pairs of cells. Note the log scale. No error bars plotted, but see (**F**). **E**. The root mean square (RMS) of the z-scores (estimated over all expectation values that were non zero in the data) during inference. **F**. The distribution of the z-scores (in blue), and in red a numerical gaussian for comparison. The RMS of these z-scores is 1.22. **G**. The number of MC samples and histogram resamples on each round during **E**.

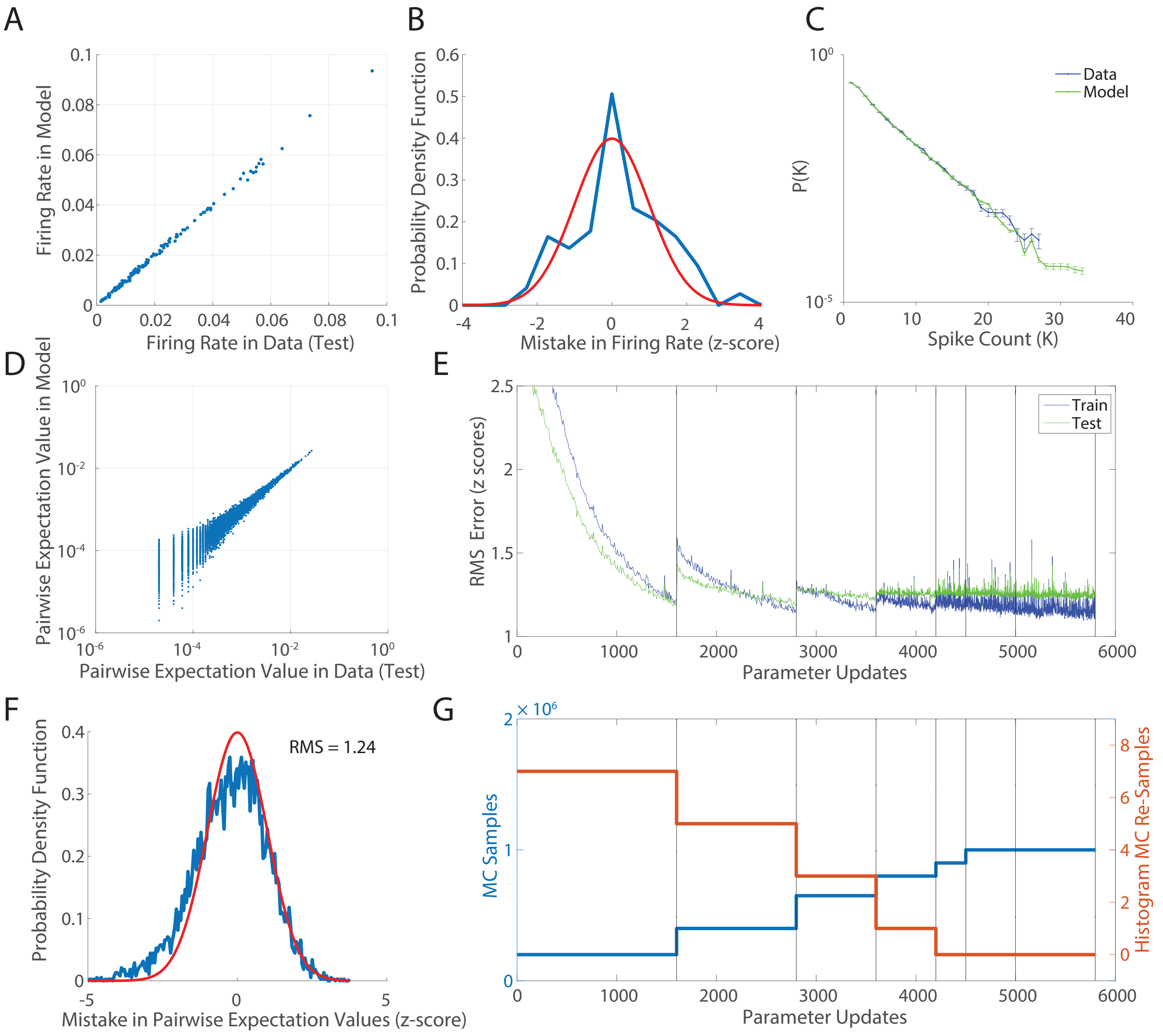

Figure S3: Quality of Fitting. Experiment #1, M1, Dark. Dataset of $N = 128$ ganglion cells recorded over a total of $2.5 \cdot 10^5$ states. The estimates of statistics in the data here use withheld (testing) data. **A**. Matching the firing rates estimated in the data with the model. These are expectation values over the binarized 20ms bins. Error bars are not shown, see panel (**B**). **B**. Distributions of the z-scores of mistakes in estimating the firing rates in panel **A**, with a numerical gaussian in red. **C**. Matching $\mathrm{P}(K)$ in the model (green) to that estimated from the data (blue). Error bars are standard error of the mean. **D**. Pairwise expectation values ($\langle r_i r_j \rangle$) estimated from the model (y-axis) plotted against the pairwise expectation values measured experimentally (x-axis), for all pairs of cells. Note the log scale. No error bars plotted, but see (**F**). **E**. The root mean square (RMS) of the z-scores (estimated over all expectation values that were non zero in the data) during inference. **F**. The distribution of the z-scores (in blue), and in red a numerical gaussian for comparison. The RMS of these z-scores is 1.24. **G**. The number of MC samples and histogram resamples on each round during **E**.

($K = \sum_{j \neq i} r_j$).

Following [Tkačik et al., 2014] we compared how well the model captured the experimental conditional probabilities (Panel B in Fig. S4,5). To do this, we took all the states in the data, and estimated the effective field for every state for every cell. We then sorted the actual binary activities by the strength of the effective field, and averaged these populations of activities in equally spaced bins (black dots). Error bars are standard deviations over the appropriate populations. These should be compared to the model prediction, which is in red (a logistic function). The gray shaded area is the probability density function of the effective field values. Inset is the same graph, on a log scale, to demonstrate that for low effective fields the conditional probabilities are well matched.

We also estimated the ability of our models to capture the probabilities of particular states. Here we compared our results to [Ganmor et al., 2011] (Panel C in Fig. S4,5). All states in the data were grouped by their unique probability (estimated directly from the data). Over these groups, we estimated the average and standard deviation of the log likelihood ratio of the model to the data. The shaded area corresponds to the 95% confidence interval given by a binary distribution.

Our model fits performed well in capturing these higher order statistics, with comparable quality to the fits in previously published results [Tkačik et al., 2014; Ganmor et al., 2011].

Analytical vs. Model Fits for Independent Networks

The analytical solution for independent neurons consists of a local field to each neuron ($h_i^{\mathrm{ind}} = -\log(\langle r_i \rangle/(1-\langle r_i \rangle))$), which depends solely on the measured firing rates ($\langle r_i \rangle$). All other terms (J_{ij}, λ_k) are zero. However, for shuffled data, inference returned models with non-zero entries in the interaction matrix, and k-potential. Because of this discrepancy we compared the shuffled data model to the analytical solution by comparing the expectation values and shapes of the specific heat predicted by both (Fig. S6).

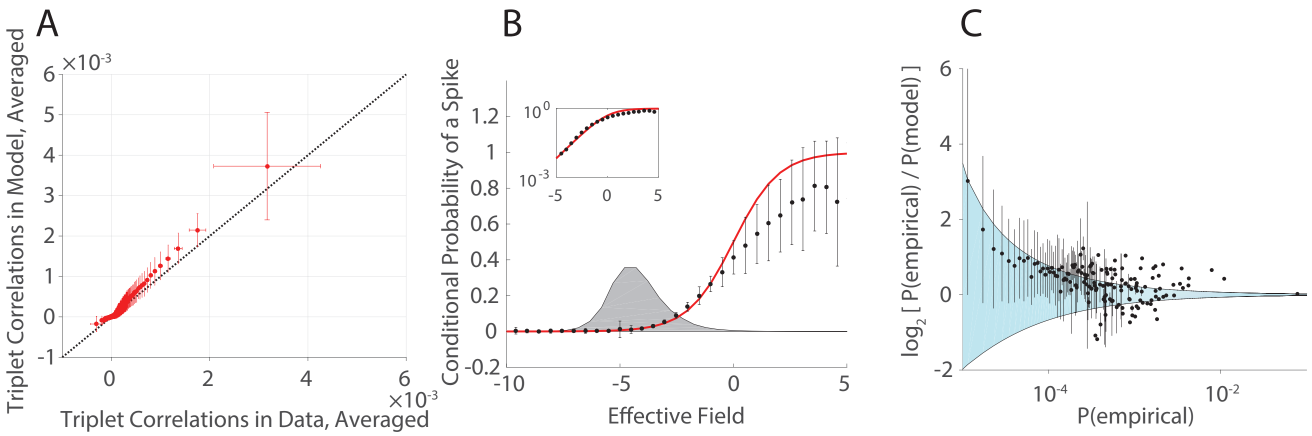

Figure S4: Matching Other Statistics in the Distribution. Experiment #1, M1, Light. Dataset of $N = 128$ ganglion cells recorded over $1.75 \cdot 10^5$ states. **A,B** follow [Tkačik et al., 2014]. **A**. Comparison of averaged triplet correlations $C_{ijk} = \langle (r_i - \langle r_i \rangle)(r_j - \langle r_j \rangle)(r_k - \langle r_k \rangle) \rangle$ in the data and the model. **B**. Comparison of model prediction of activity (based on effective fields) to the actual occurences of spikes in the data. **C**. Following [Ganmor et al., 2011] we estimated how well the model captures the probabilities of states occuring in the data (empirical). The shaded area corresponds to the 95% confidence interval given by a binary distribution.

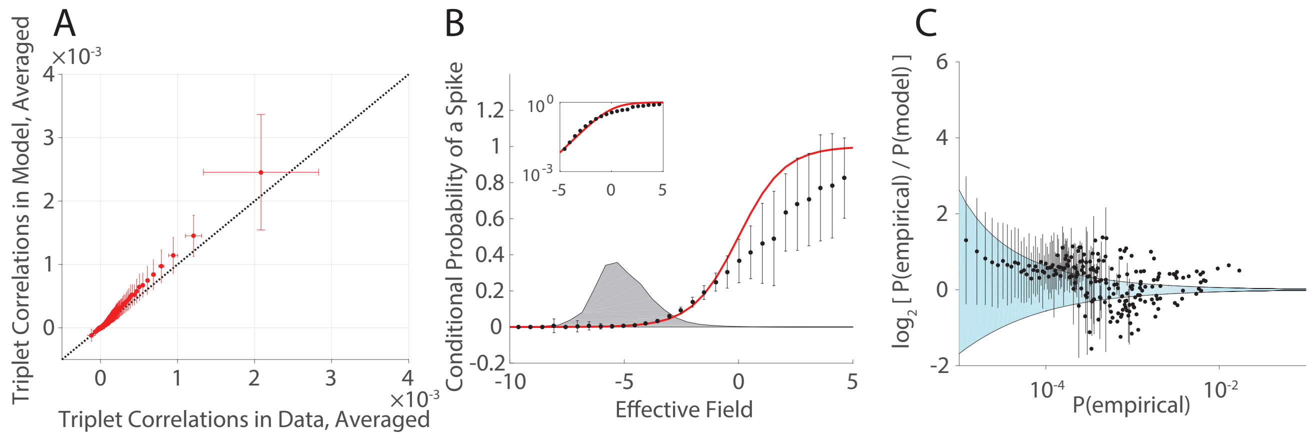

Figure S5: Matching Other Statistics in the Distribution. Experiment #1, M1, Dark. Dataset of $N = 128$ ganglion cells recorded over $2.5 \cdot 10^5$ states. **A,B** follow [Tkačik et al., 2014]. **A**. Comparison of averaged triplet correlations $C_{ijk} = \langle (r_i - \langle r_i \rangle)(r_j - \langle r_j \rangle)(r_k - \langle r_k \rangle) \rangle$ in the data and the model. **B**. Comparison of model prediction of activity (based on effective fields) to the actual occurences of spikes in the data. **C**. Following [Ganmor et al., 2011] we estimated how well the model captures the probabilities of states occuring in the data (empirical). The shaded area corresponds to the 95% confidence interval given by a binary distribution.

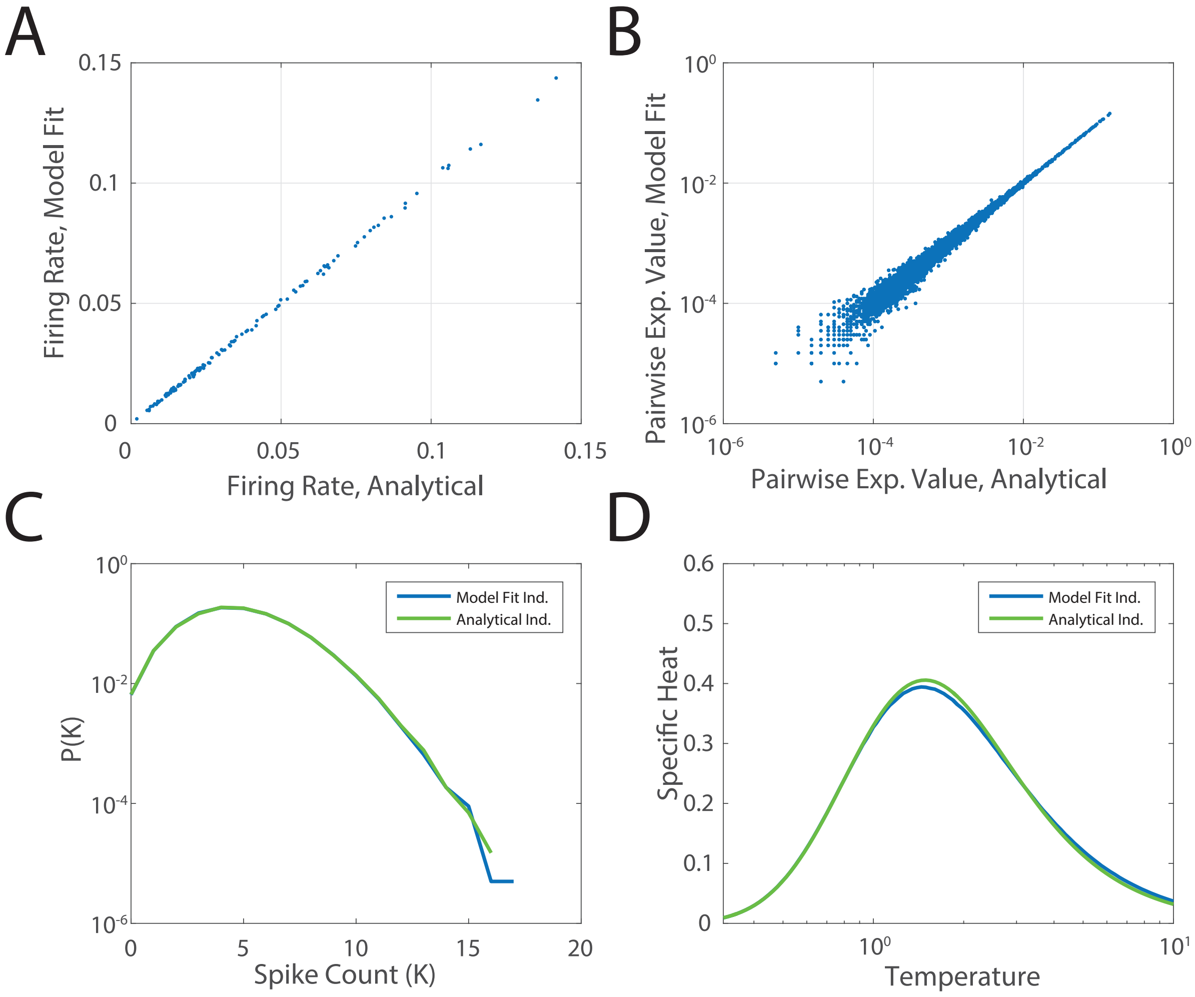

Figure S6: Comparing Analytical Solutions to Shuffled-Data Model Fits. For the analytical solution, we took all $(1.75 \cdot 10^5)$ of the states in the *light* condition , to estimate the average activity of each neuron. For the shuffled data, model parameters were inferred on a training subset of all of those spikes, as described earlier in the Supplement. **A.** Firing rates (estimated over 20ms time bins) compared across the two models, for $N = 128$ cells in the data. **B.** Pairwise expectation values ($\langle r_i r_j \rangle$, estimated over 20ms binarized bins) compared across the two models. **C.** Distribution of Spike Counts, $P(K)$, for the two models. **D.** Specific heat as a function of temperature, for the two models.

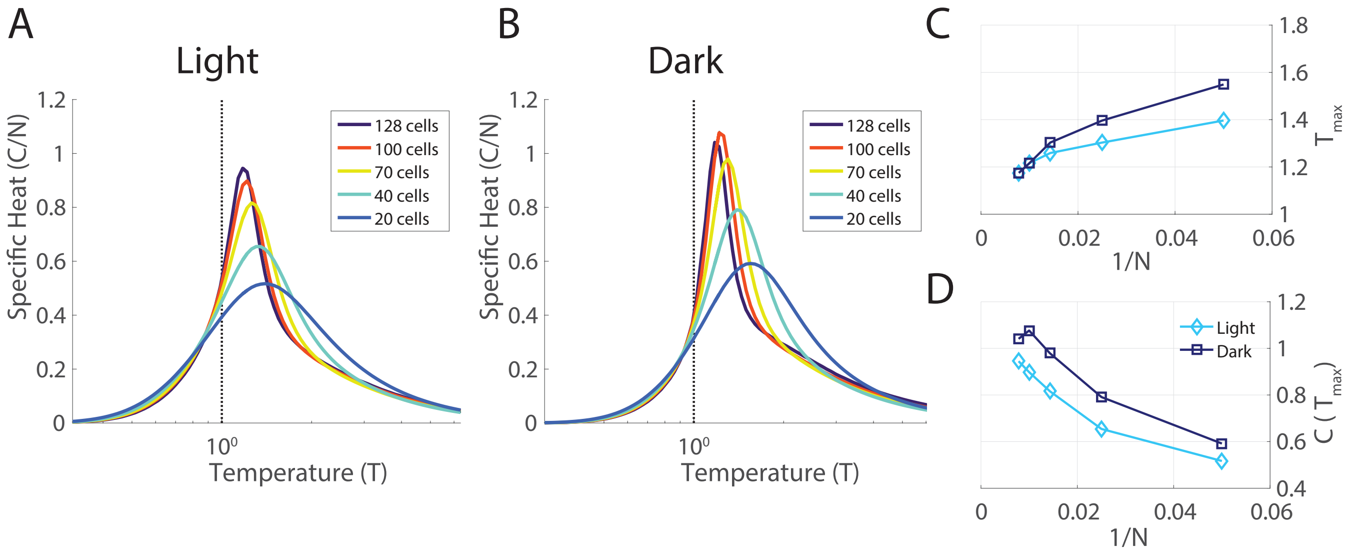

Figure S7: Pairwise Maximum Entropy Model Results for Natural Movies (Experiment #1, M1, *light* and *dark*, $N = 128$ ganglion cells). All manipulations are identical to those done in the main text. **A**. The specific heat for subsampled populations in the *light* condition. **B**. The specific heat for subsampled populations in the *dark* condition. **C,D**. The peak temperature, T_{max}, and peak value, $C(T_{max})$, of the specific heat are plotted as a function of the inverse size of the analyzed neural population, $1/N$.

Pairwise Maximum Entropy Model Results

Our main result concerning the presence of a phase transition was qualitatively similar in pairwise maximum entropy model fits to the data. As in the main text, the pairwise maximum entropy model was fit to subsets of cells in the *light* and *dark* datasets. A fictitious temperature was introduced, and the variance of energy levels was evaluated over Monte Carlo samples from these distributions. The specific heat at different system sizes for the *light* dataset is plotted in Fig. S7A, for the *dark* in Fig. S7B. The peak temperature, T_{max}, and peak value, $C(T_{max})$, of the specific heat are plotted as a function of the inverse size of the analyzed neural population, $1/N$ (Fig. S7C,D). As for the k-pairwise model, the peak sharpens, yet is always to the right of the operating point of the network, suggesting that the network is on the low-temperature side of the phase transition.

Characterizing the Transition With Respect to Correlation, α

We found a transition in the properties of inferred networks while scaling the correlation strength down by a constant α, with α ranging from 0 to 1 ($N = 128$ curve in Figure 7C). To understand this transition further, we subsampled networks of different system size, inferred pairwise maximum entropy models for these subnetworks of neurons, and calculated the specific heat of each subnetwork (Figure 7C, Figure S8A). When we plotted the specific heat as a function of correlation strength for different subnetwork sizes, we found that the shapes of all of the curves appeared qualtatively similar (Fig. S8A). This observation suggested that a relatively simple scaling relationship for the contribution to the specific heat that depended on the system size might be able to account for this set of different curves.

To estimate this N-dependent scaling, we subtracted out the specific heat for subnetworks with $N = 20$ cells from all the other larger networks, and asked how this change in the specific heat ($\Delta C_N = C_N - C_{N=20}$) scaled with the relative change in system size ($N - 20$) (Fig. S8B). For the fully correlated system ($\alpha = 1$), we found a power law relationship between the change in the specific heat, ΔC_N, and the change in the system size, ($N - 20$), with an exponent of $\gamma = 0.65$ (95% confidence bounds: 0.58 and 0.71). We then found that changes in the specific heat at other values of α collapse if scaled by this power law. In other words, the quantity $\Delta C_N/(N - 20)^\gamma$ had a similar shape for the different values of N (Fig. S8C). The shape of the specific heat above some critical point (α^*) could be described as having an additional sigmoidal term whose amplitude had a N-dependence, but which was mostly unchanged across system size.

We write mostly because the scaled curves in Fig. S8C did exhibit some minor systematic dependence on system size N. There were two possibilities here: First, that the contribution to the specific heat above α^* vanishes at α^*. This would indicate a discontinuity in the derivative of the specific heat, classifying the phase transition as third-order. Second, the contribution to the specific heat is finite, providing a jump at the critical point α^*. Such a discontinuity would classify the phase transition as second-order. We applied finite-size extrapolation to help disambiguate between the two possibilities.

To do this, we fit the scaled change in the specific heat to the inverse of the scaled change in system size for the three largest networks ($N = 70, 100, 128$), for a given value of the correlation, α. The intercept of such a fit is the extrapolated estimate of the behavior of the specific heat at the thermodynamic limit (as $N \to \infty$). An example of this extrapolation for $\alpha = 0.34$ is plotted in Fig. S8D. When we plotted the extrapolated values of the specific heat ($N \to \infty$) as a function of the correlation strength, we found that this curve again appeared to be sigmoidal (Fig. S8E). This suggests that the term in the specific heat that depends on system size vanishes at the critical point, which is more consistent with a third-order phase transition than a second-order one.

To estimate the critical point, we fit the finite-size extrapolation with a hyperbolic tangent truncated at the halfway point: all values less than α^* set to zero (Matlab's `nlinit` function, dashed line in Fig. S8E). Such a function is continuous but its derivative is discontinuous at α^*. This provided us with an estimate of the critical point, $\alpha^* = 0.232$.

To summarize, the extrapolated behavior of the specific heat in the thermodynamic limit was similar in shape to the measured behavior of the full system with $N = 128$ neurons. Thus, extrapolating did not change the qualitative nature of the result: this phase transition remained more consistent with third-order than second-order.

Persistence Indices

We saw in the main text that the persistence index for any given neural activity state correlated well with its dwell time estimated by finite temperature ($T = 1$) MC sampling dynamics (Fig. 9C).

We show here a plot of the persistence indices from the same neural activity state measured in the light vs dark conditions, which exhibited high correlation (Fig. S9A). This correlation is evidence that the energy landscape has a similar shape in light and dark conditions, even though the specific details of the neural code change considerably (Fig. 2). This similarity is important, because the stimulus was the same in both conditions. We expect that the retina should encode many of the same stimulus features across different light levels. One possibility is that the retina uses different codewords across different light levels, in which case the brain must change its decoding strategy as

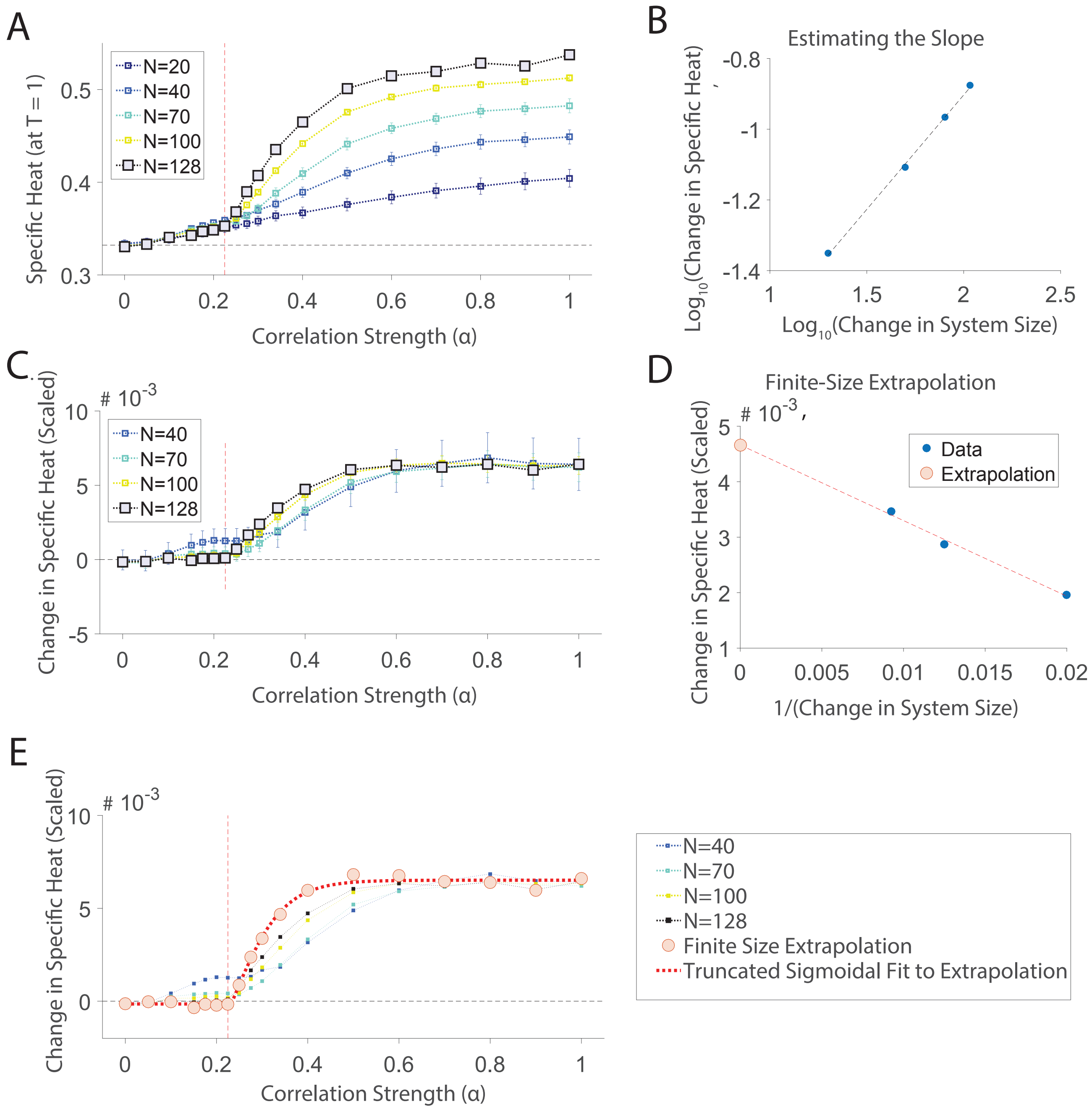

Figure S8: Characterizing the Transition with Respect to Correlation

A. Specific heat plotted as a function of the correlation strength, α, for different choices of system size, N (different colors). (Note: This panel is identical to Fig. 7C). **B**. To estimate the N-dependence of the specific heat, we subtracted out the specific heat of the $N = 20$ cell network from the larger networks, to calculate $\Delta C_N = C_N - C_{(N=20)}$. Here, ΔC_N (for $\alpha = 1$) is plotted vs. the change in system size $(N - 20)$, on a log-log scale. The linear fit had a slope of $\gamma = 0.65$. **C**. The curves of the scaled change in the specific heat, $\Delta C_N/(N-20)^\gamma$, plotted as a function of correlation strength for different values of the system size, N (squares). **D**. An example of the extrapolation performed per α (here $\alpha = 0.34$): $\Delta C_N/(N-20)^\gamma$ plotted as a function of the change in system size $(N-20)$. The intercept provides an estimate of the $N \to \infty$ value. **E**. The scaled change in the specific heat, plotted as a function of correlation strength α and system size N (squares), as well as the extrapolated value (red circles).

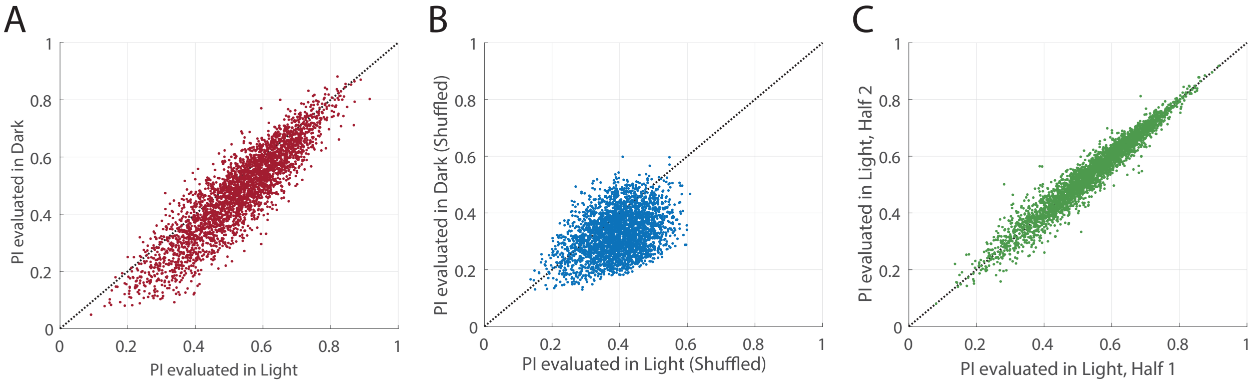

Figure S9: Persistence Indices. Here, we compare the persistince indices for the 3187 states from the *light* natural movie recording with $K = 12$, using different model parameters. **A**. PI estimated by the full model fitting the natural movie in the *light* (x-axis) and *dark* (y-axis) adapted conditions (correlation coefficient of 0.90). **B**. PI estimated by the independent model (shuffled data) fitting the natural movie in the *light* (x-axis) and *dark* (y-axis) adapted conditions (correlation coefficient of 0.37). **C**. PI estimated from model fits to two complementary random halves of the *light* dataset (correlation coefficient of 0.97).

a function of the light level. The other possibility is that some aspects of the retinal code remains invariant across light levels. While no such invariance was obvious at the level of single neurons (see Fig. 1 and [Tikidji-Hamburyan et al., 2015]), these data provide the first evidence that a form of light-level invariance does exist at the population level.

For comparison, we also plot the persistence indices estimated from independent models in the *light* and *dark* adapted conditions (Fig. S9B). Here, the correspondence between the neural code in light and dark conditions was poor, with a correlation coefficient = 0.37 in Fig. S8B vs 0.90 in Fig. S9A.

Clearly, the form of invariance to light level that we observed in the population neural code (Fig. S9A) is a consequence of the correlations in the experiment. To test the reproducibility of this measure, we split the *light* data into two random, complementary halves, and inferred the k-pairwise maximum entropy model parameters for these halves two separately. The persistence indices display a high degree of similarity across these two random halves (Fig. S9C), indicating that this a highly reproducible measure that can be well sampled in our data. The comparison with Fig. S9A also shows that the invariance of the population code to light level nearly approaches the limit set by sampling noise.

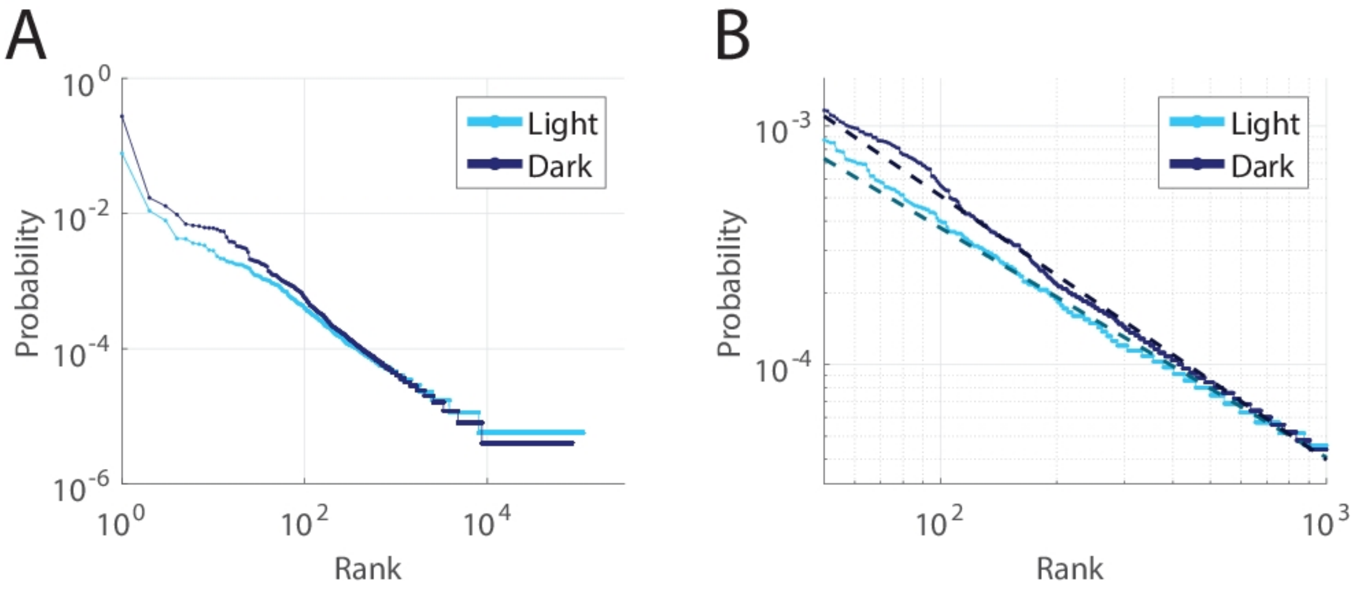

Figure S10: Zipf Relationships. Probability vs. Rank for States from the *light* (light blue) and *dark* (dark blue) datasets evaluated directly. On the left, all states are plotted for both datasets. On the right, the probabilities of the states ranked from 50 to 1000 only are plotted, and a linear fit to this subset of states is shown in dashed lines. The fitted slopes are -0.96 for the *light* and -1.11 for the *dark* datasets.

Zipf-like Relationships in the Distribution of Codewords

A separate line of work has investigated the presence of Zipf-like relationships in the probability distribution of neural codewords [Mora & Bialek, 2011; Schwab et al., 2014]. We largely avoided working with these quantities out of concerns about the adequacy of sampling the we could achieve. In Fig. S10 we plot the Zipf relationship between the probability and rank of a state (estimated directly from the natural movie dataset in Experiment #1). While the slopes of a power law fit to our experimental data were close to -1, it is important to note that a clear power law relationship existed only over a small range of rank $\sim$ 200 to 1000. This leaves a substantial uncertainty in the estimate of the slope. But more fundamentally, we are not sure how to interpret deviations from a slope of -1 as well as how to think about deviations from the power law form itself, such as the "bump" near rank $\sim$ 70 in the dark and near rank $\sim$ 300 in the light (Fig. S10).

Nonlinear Susceptibility

We calculated the nonlinear susceptibility for the retinal population code in the *light*. This quantity is defined as (see Chpt. 2.5 in [Fischer & Hertz]):

$$\chi^{(nl)} = \frac{2}{N(N-1)T^3} \sum_i \sum_{j<i} \left(\langle r_i r_j \rangle - \langle r_i \rangle \langle r_j \rangle \right)^2 \tag{5}$$

Where the inner brackets denote averages over $(2.5 \cdot 10^5)$ monte carlo samples at the given temperature. The nonlinear susceptibility is a higher-order derivative of the free energy. This quantity is typically studied in spin glass models, where a peak in $\chi^{(nl)}$ indicates a spin glass phase transition at finite size. We evaluated this term in the pairwise maximum entropy models corresponding to the retinal population code in the *light*, as a function of system size N and temperature T (Fig. S11 A).

When the nonlinear susceptibility is divergent as it is near the freezing transition in a spin glass (a high-order critical point), the value of the nonlinear susceptibility at a given temperature increases with increasing system size. Because the nonlinear susceptibility is the square of the correlation, and the correlations can only increase as larger system sizes are considered (near a critical point of this type), the value of the nonlinear susceptibility grows everywhere in the vicinity of such a critical point (see for example, [Aspelmeier et al., 2016]).

What we observe is that the nonlinear susceptibility decreases with system size for $T < 1$ (see Fig. S11 A). At $T = 1$, the inference constraint on correlations means that the nonlinear susceptibilities are all equal. As $T > 1$, there is a large peak in the nonlinear susceptibility that increases and sharpens with system size. Somewhere around $T = 1.5$ the nonlinear susceptibility of the full, $N = 128$ neuron network falls below the nonlinear susceptibility of the $N = 100$ and $N = 70$ neuron networks (see Fig. S11 B). This is not the dependence on system size that one would observe near a spin glass critical point. Similar cross-overs were seen at higher temperatures – for instance the nonlinear susceptibility for $N = 70$ drops below that of $N = 40$ around $T = 1.9$. The fact that these intersections occur at different locations in temperature means that there is no

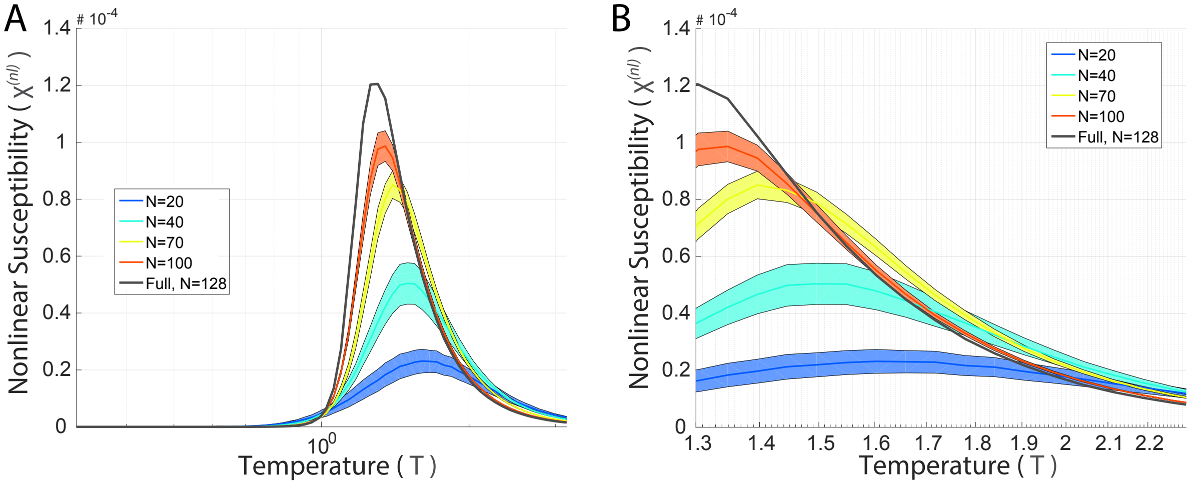

Figure S11: Nonlinear susceptibility. The nonlinear susceptibility is evaluated here during an annealing process (same as for the specific heat calculations), for the networks of different system size N, taken from the *light* condition. Error bars are standard error of the mean, evaluated over ten different networks of the same size N, as in the specific heat calculations earlier. **B** is the same as **A**, magnified along the horizontal axis.

single point at which the nonlinear susceptibility starts to decrease with system size. That rules out the existence of a second phase transition at any higher temperature. Finally, this type of behavior in the nonlinear susceptibility is very similar to the sharpening that was observed in the specific heat, which can be interpreted to mean that no additional discontinuities were found at these higher order derivatives of the free energy. This evidence is then again consistent with the underlying phase transition being first-order.

References

Aspelmeier, T., Katzgraber, H. G., Larson, D., Moore, M. A., Wittmann, M., & Yeo, J. (2016). Finite-size critical scaling in Ising spin glasses in the mean-field regime. *Physical Review E, 93*(3), 032123.

Baylor, D. A., Nunn, B. J., & Schnapf, J. L. (1987). Spectral sensitivity of cones of the monkey Macaca fascicularis. *The Journal of Physiology, 390*, 145.

Broderick, T., Dudik, M., Tkacik, G., Schapire, R. E., & Bialek, W. (2007). Faster solutions of the inverse pairwise Ising problem. *arXiv preprint arXiv:0712.2437.*

Cornwall, M. C., MacNichol, E. F., & Fein, A. (1984). Absorptance and spectral sensitivity measurements of rod photoreceptors of the tiger salamander, Ambystoma tigrinum. *Vision research, 24*(11), 1651-1659.

Dudik, M., Phillips, S. J., & Schapire, R. E. (2004). Performance guarantees for regularized maximum entropy density estimation. In *Learning Theory* (pp. 472-486). Springer Berlin Heidelberg.

Fischer, K. H., & Hertz, J. A. (1993). *Spin glasses* (Vol. 1). Cambridge university press

Ganmor, E., Segev, R., & Schneidman, E. (2011). Sparse low-order interaction network underlies a highly correlated and learnable neural population code. *Proceedings of the National Academy of Sciences, 108*(23), 9679-9684.

Hertz, J., Krogh, A., & Palmer, R. G. (1991). *Introduction to the theory of neural computation* (Vol. 1). Basic Books.

Kirkpatrick, S., & Sherrington, D. (1978). Infinite-ranged models of spin-glasses. *Physical Review B, 17*(11), 4384.

Loback, A.R., Prentice, J.S., Ioffe, M.L., & Berry, M.J. (2016). Noise-robust modes of the retinal population code geometrically correspond with "ridges". In prep.

Macke, J. H., Opper, M., & Bethge, M. (2011). Common input explains higher-order correlations

and entropy in a simple model of neural population activity. *Physical Review Letters, 106*(20), 208102.

Mora, T., & Bialek, W. (2011). Are biological systems poised at criticality?. *Journal of Statistical Physics, 144*(2), 268-302.

Palmer, R. G. (1982). Broken ergodicity. *Advances in Physics, 31*(6), 669-735.

Perry, R. J., & McNaughton, P. A. (1991). Response properties of cones from the retina of the tiger salamander. *The Journal of Physiology, 433*, 561.

Schneidman, E., Berry, M. J., Segev, R., & Bialek, W. (2006). Weak pairwise correlations imply strongly correlated network states in a neural population. *Nature, 440*(7087), 1007-1012

Schwab, D. J., Nemenman, I., & Mehta, P. (2014). Zipf's law and criticality in multivariate data without fine-tuning. *Physical review letters, 113*(6), 068102.

Sherry, D. M., Bui, D. D., & Dgerip, W. J. (1998). Identification and distribution of photoreceptor subtypes in the neotenic tiger salamander retina. *Visual neuroscience, 15*(06), 1175-1187.

Tholence, J. L. (1980). On the frequency dependence of the transition temperature in spin glasses. *Solid State Communications, 35*(2), 113-117.

Tikidji-Hamburyan, A., et al. (2015). Retinal output changes qualitatively with every change in ambient illuminance. *Nature neuroscience, 18* (1), 66-74.

Tkačik, G., Schneidman, E., Berry, M.J., & Bialek, W. (2006). Ising models for networks of real neurons. *arXiv preprint* q-bio/0611072.

Tkačik, G., et al. (2014). Searching for collective behavior in a large network of sensory neurons. *PLoS Comput Biol, 10*(1), e1003408.
